# Supplementary material for: Predicting response to immunotherapy in gastric cancer via multi-dimensional analyses of the tumour immune microenvironment
Source: Nat Commun. 2022 Aug 18;13:4851. doi: 10.1038/s41467-022-32570-z (PMC9388563; doi:10.1038/s41467-022-32570-z)
Supplement: Supplementary file 1 — Supplementary Information [file 41467_2022_32570_MOESM1_ESM.pdf]

Supplementary Information

Predicting response to immunotherapy in gastric cancer via multi-dimensional analyses of the tumour immune microenvironment

Chen et al.

Supplementary Figures

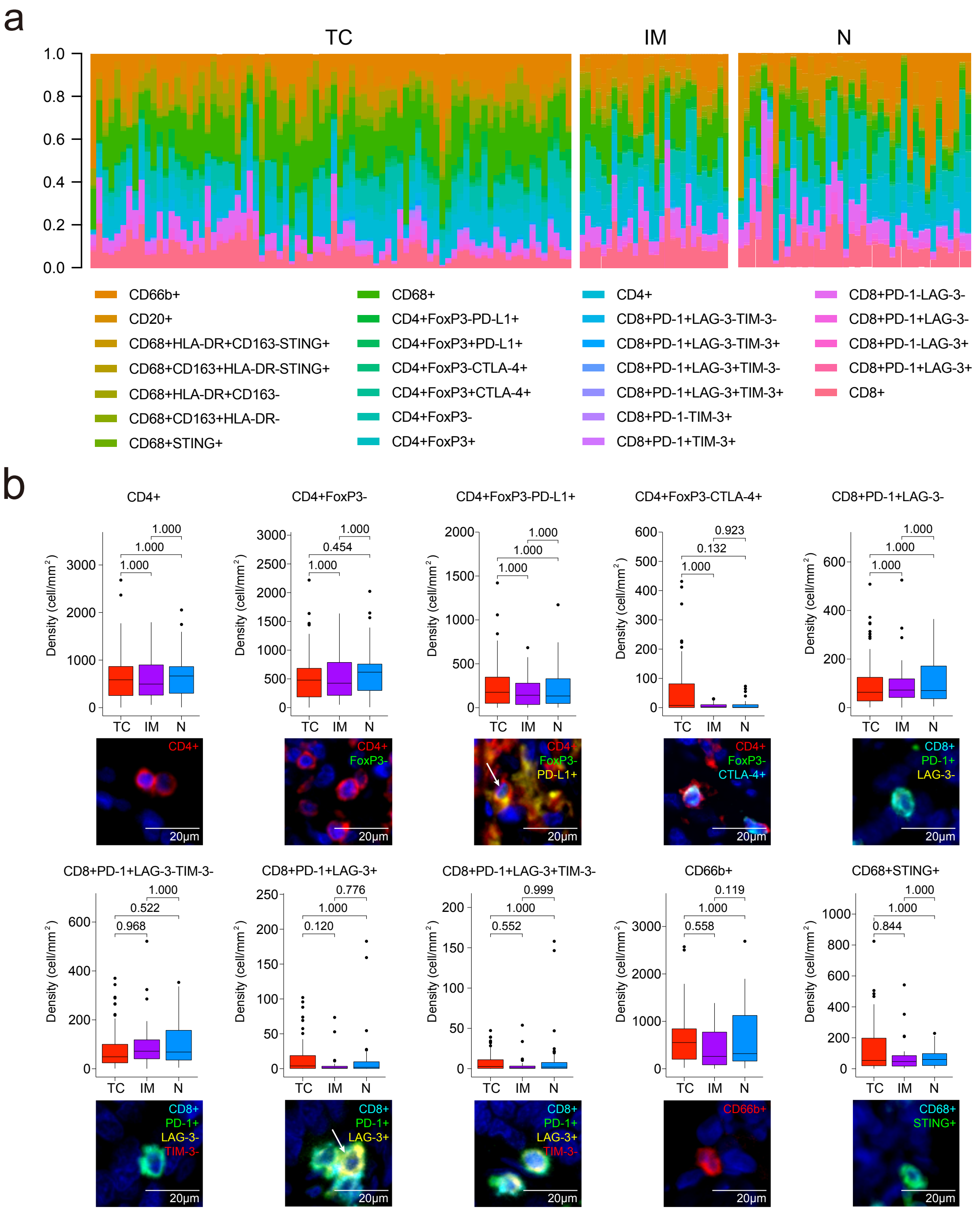

Supplementary Figure 1. The immune composition of GC specimens.

(a) Relative abundance of 26 TIIC populations in the context of different regions of interest (ROIs). TC, tumour core; IM, invasion margin; N, normal.

(b) Distinct quantification of the density of the different TIIC populations across the ROIs (n = 80). The immunofluorescence staining images represent the co-expression of the corresponding markers and DAPI (nuclei). Scale bar: 20 µm. Box and whiskers represent mean ± 10–90 percentile. Kruskal–Wallis test with the Dunn multiple comparison.

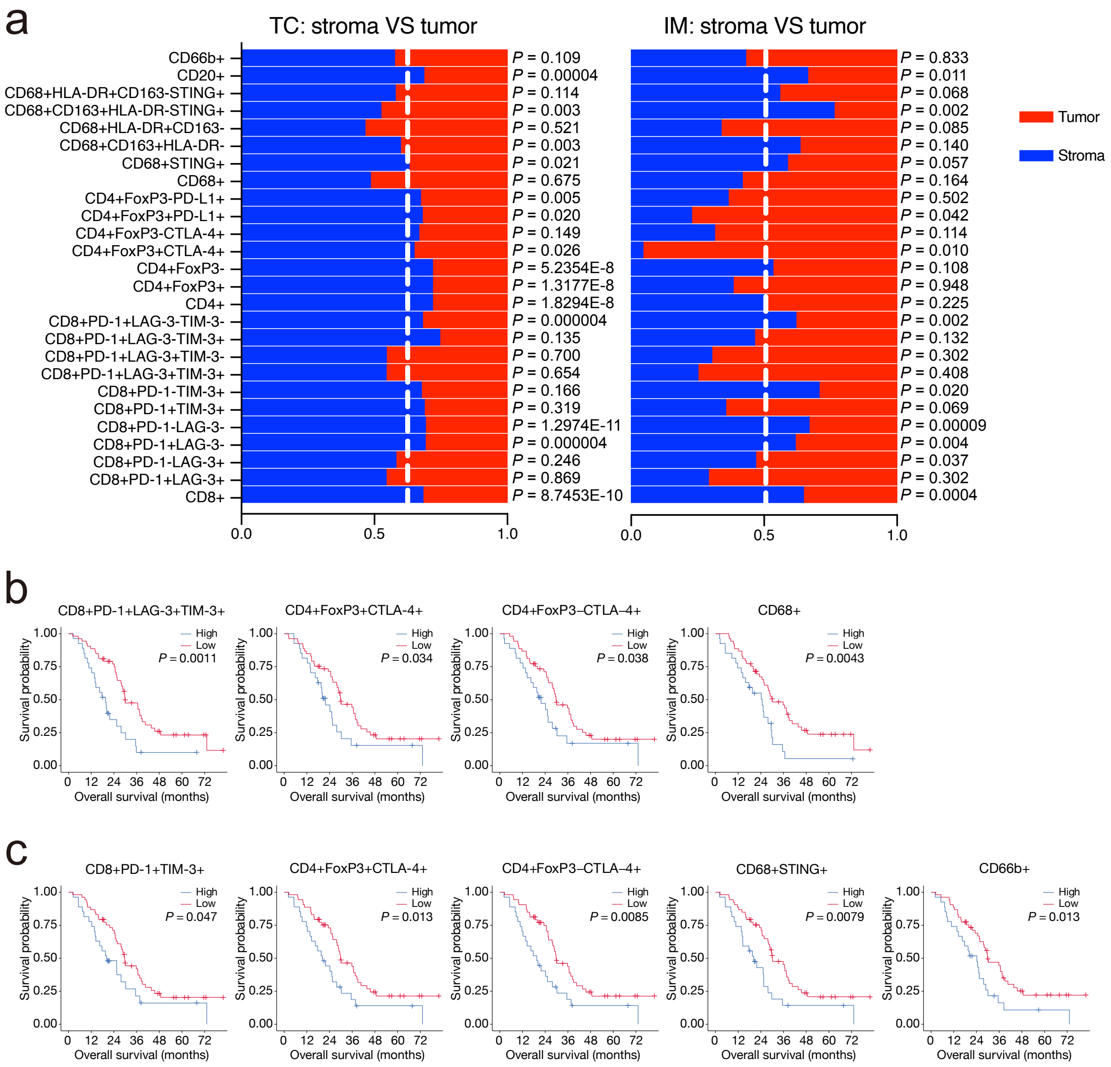

Supplementary Figure 2. The immune composition in the tumour *versus* stromal tissues.

(a) Comparison of density of the 26 TIIC populations between the tumour and stroma in the tumour core and invasion margin. Statistical relevance was assessed using the two-sided Mann-Whitney *U* test.

The overall survival of the 80 patients based on the density of TIICs in the tumour (b) and stroma (c) is represented. Individual immune infiltrate values were divided into high (> 2/3 of the patients in the cohort; blue line) or low density (≤ 2/3 of patients in the cohort; red line). Statistical relevance was assessed using the Log-rank (Mantel-Cox) test.

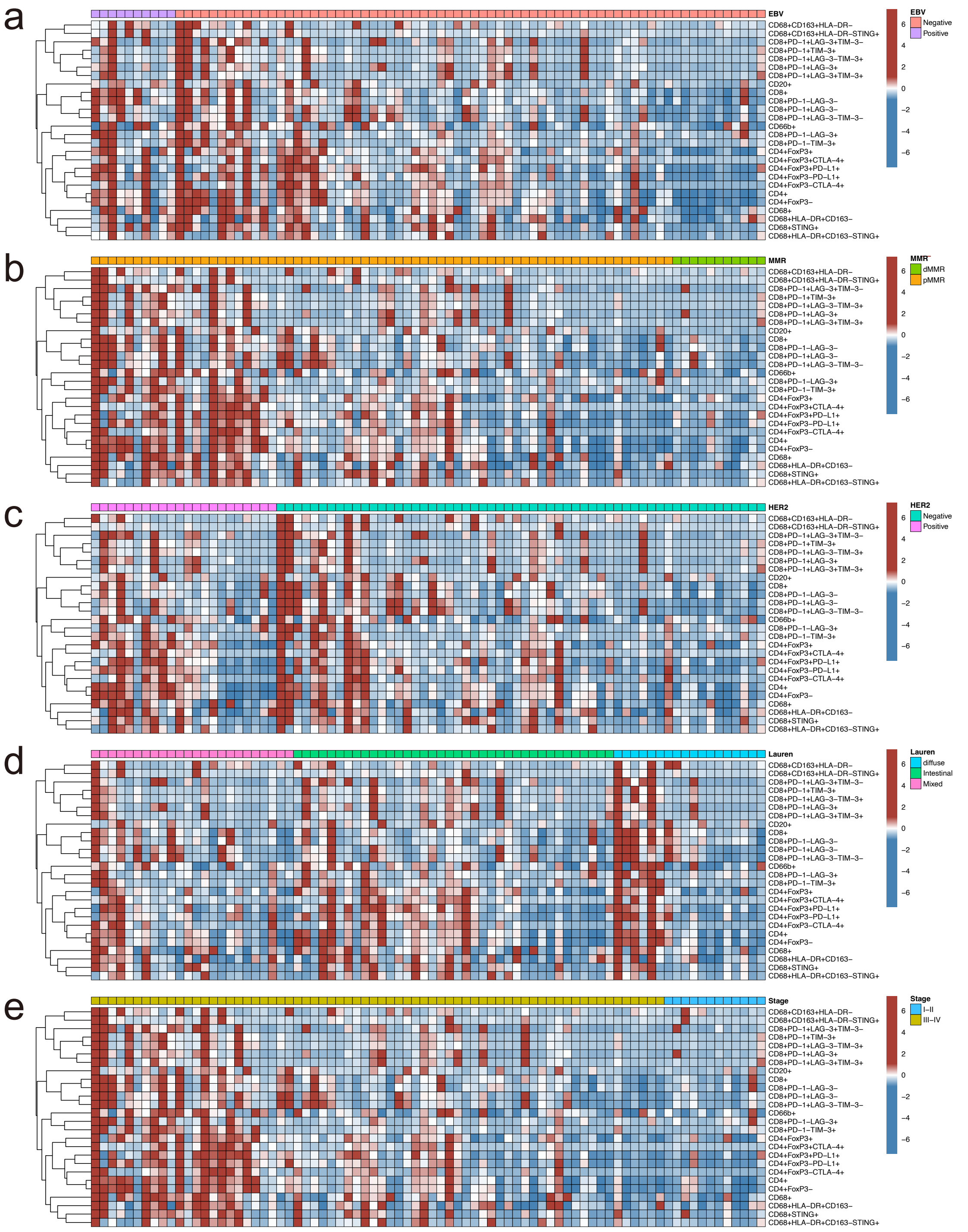

Supplementary Figure 3. The heatmap of immune composition in GC.

TIIC density grouped by EBV status (a), MMR status (b), HER2 expression (c), Lauren classification (d), and stage (e).

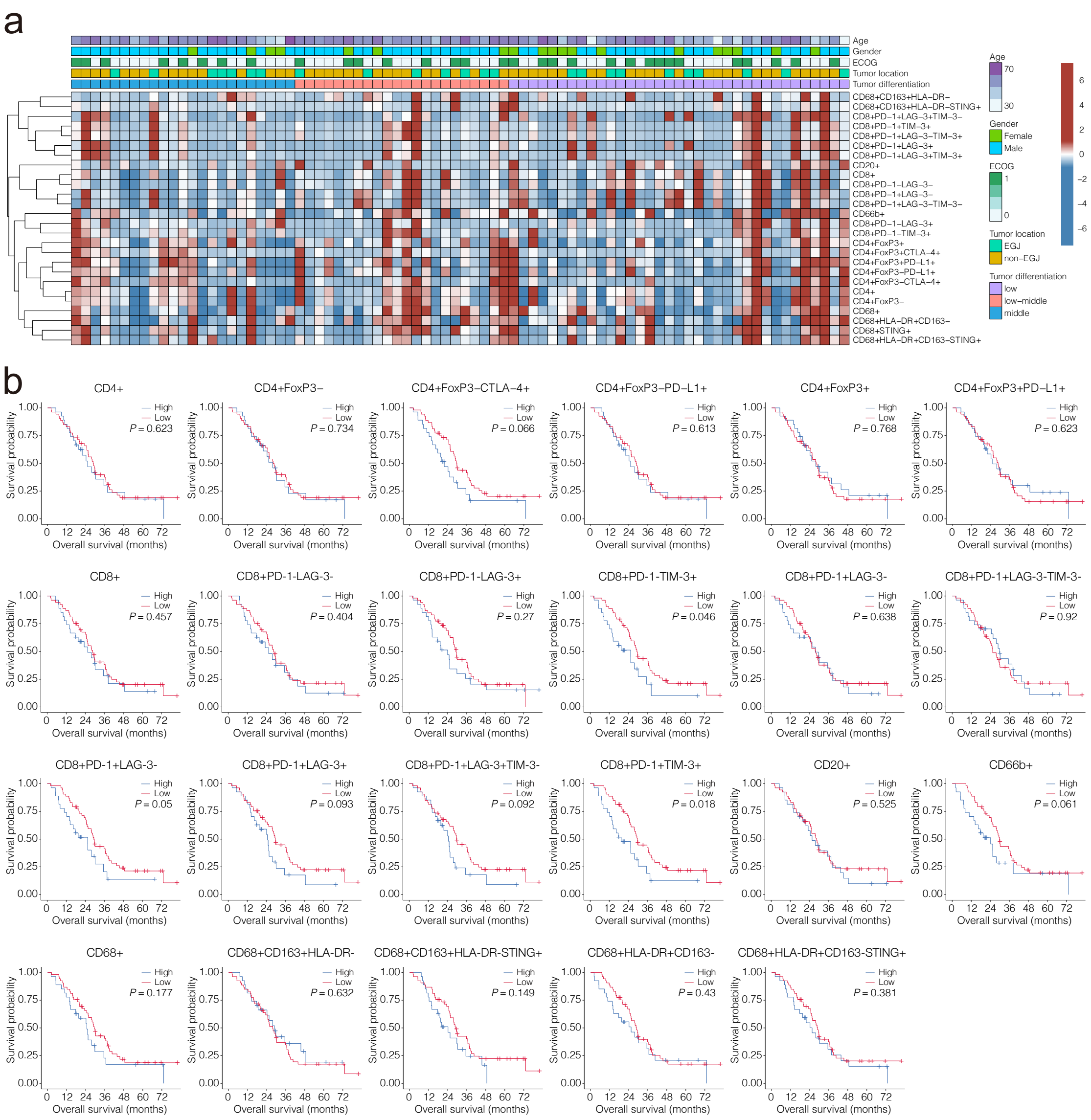

Supplementary Figure 4. Immune composition and survival analysis.

(a) TIIC density grouped by GC subtype (age, sex, ECOG status, tumour location, and tumour differentiation).
(b) Overall survival of the 80 patients based on the density of TIICs. The individual immune infiltrate values were divided into high (> 2/3 of the patients in the cohort; blue line) or low density (≤ 2/3 of patients in the cohort; red line). Statistical relevance was assessed using the Log-rank (Mantel-Cox) test.

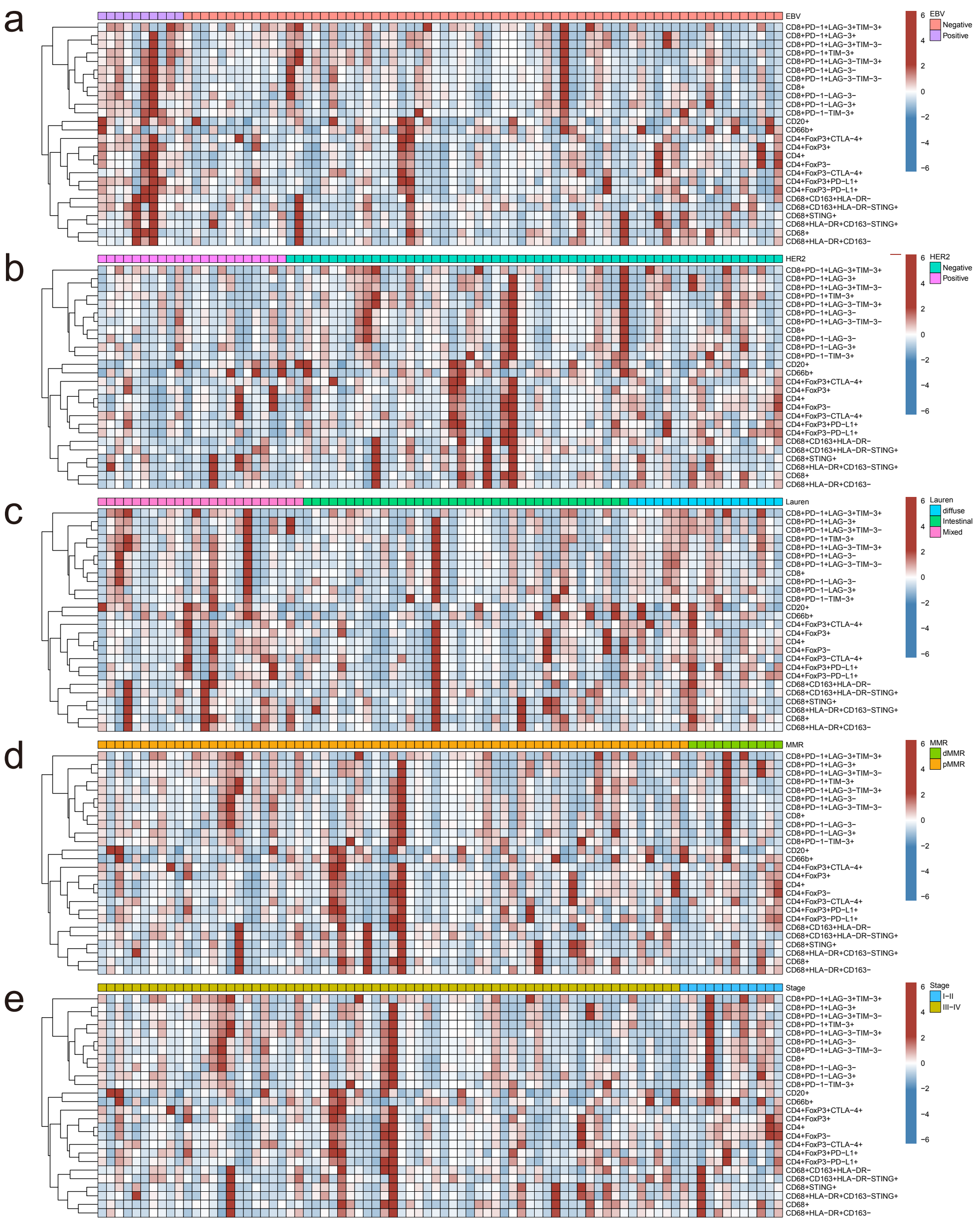

Supplementary Figure 5. The heatmap of spatial analysis of cell distribution in GC.

Effective score of TIICs in patients grouped by EBV status (a), HER2 expression(b), Lauren classification (c), MMR status (d), and stage (e).

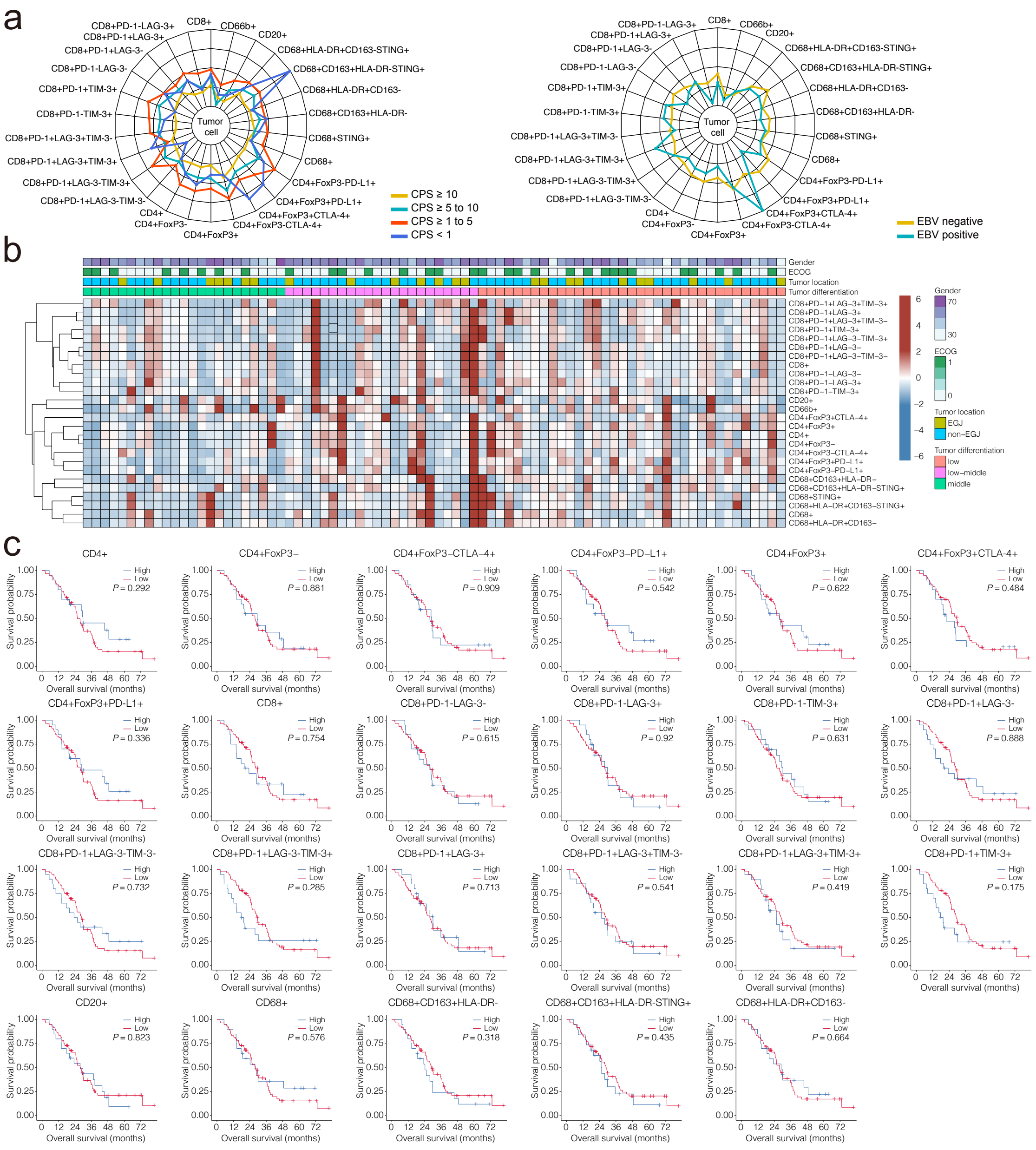

Supplementary Figure 6. Spatial analysis of cell distribution in GC and survival analysis.

(a) Median distance between TIICs and tumour cells depending on the CPS score and EBV status.
(b) Effective score of TIICs in patients grouped by GC subtypes (age, gender, ECOG, tumour location, tumour differentiation).
(c) Overall survival of the 80 patients based on the effective density (0-20 μm) of TIICs. The individual immune infiltrate values were divided into high (> 2/3 of the patients in the cohort; blue line) or low density (≤ 2/3 of patients in the cohort; red line). Statistical relevance was assessed using the Log-rank (Mantel-Cox) test.
ETC, extra tree classifier; GBC, gradient boosting classifier; ABC, AdaBoost classifier; MLP, multilayer perceptron.

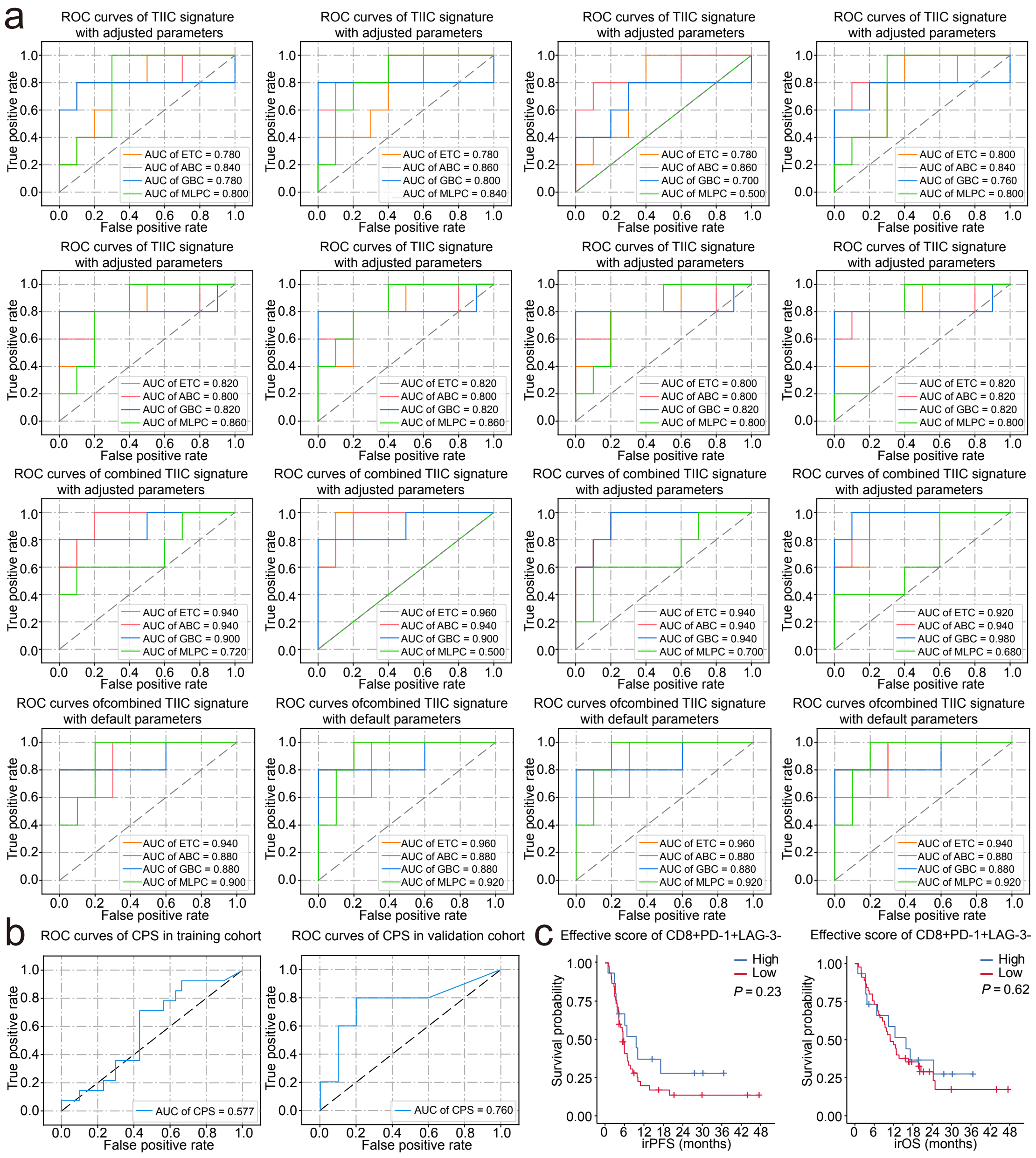

Supplementary Figure 7. TIIC-signature predicts the response to immunotherapy.

(a) Representative receiver operating characteristic (ROC) curves for the performance of the identified TIIC-signature and combined TIIC-signature in GC patients subjected to immunotherapy in validation cohorts.
(b) ROC curves for the performance of the CPS score in GC patients subjected to immunotherapy in the training and validation cohorts.
(c) Kaplan-Meier curves of the irPFS and irOS of spatial marker. Statistical relevance was assessed using the Log-rank (Mantel-Cox) test.

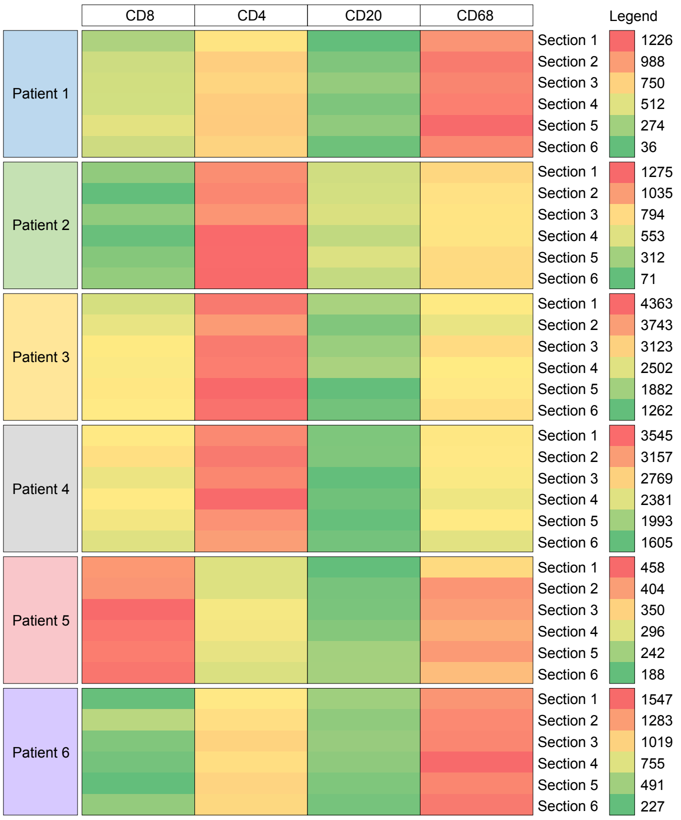

Summary of coefficient of variation

ID	CD8	CD4	CD20	CD68
Patient 1	0.14	0.14	0.41	0.10
Patient 2	0.29	0.13	0.08	0.10
Patient 3	0.05	0.09	0.07	0.08
Patient 4	0.04	0.07	0.02	0.02
Patient 5	0.03	0.04	0.13	0.04
Patient 6	0.06	0.16	0.03	0.11

Supplementary Figure 8. The densities of 4 major cell types (CD4+ T cells, CD8+ T cells, B cells, and macrophages; represented by CD4, CD8, CD20, and CD68, respectively) in six samples (six sections for each sample)

Supplementary Table 1. The distribution of tumour-infiltrating immune cells density in tumour core across different stages

	Stage		
	I-II (N = 12)	III-IV (N = 68)	P value
$CD8^+$	243.9 ± 175.5	415.4 ± 361.4	0.051
$CD8^+PD\text{-}1^+LAG\text{-}3^-$	44.9 ± 27.7	105.3 ± 107.3	0.085
$CD8^+PD\text{-}1^+LAG\text{-}3^+$	7.0 ± 12.5	15.5 ± 24.0	0.37
$CD8^+PD\text{-}1^-LAG\text{-}3^+$	9.9 ± 14.7	14.4 ± 25.5	0.74
$CD8^+PD\text{-}1^-LAG\text{-}3^-$	182.1 ± 146.9	280.1 ± 254.0	0.095
$CD8^+PD\text{-}1^+TIM\text{-}3^+$	5.0 ± 11.0	24.9 ± 47.4	0.072
$CD8^+PD\text{-}1^-TIM\text{-}3^+$	10.9 ± 16.5	42.9 ± 72.4	0.049
$CD8^+PD\text{-}1^+LAG\text{-}3^+TIM\text{-}3^-$	4.9 ± 11.0	8.4 ± 11.3	0.34
$CD8^+PD\text{-}1^+LAG\text{-}3^+TIM\text{-}3^+$	2.1 ± 4.8	7.1 ± 14.4	0.51
$CD8^+PD\text{-}1^+LAG\text{-}3^-TIM\text{-}3^+$	2.9 ± 6.3	17.8 ± 36.2	0.028
$CD8^+PD\text{-}1^+LAG\text{-}3^-TIM\text{-}3^-$	42.0 ± 26.2	87.5 ± 87.9	0.15
$CD4^+$	311.3 ± 263.2	706.4 ± 521.5	0.006
$CD4^+FoxP3^-$	230.9 ± 209.8	566.5 ± 425.0	0.006
$CD4^+FoxP3^+$	80.4 ± 71.7	139.9 ± 114.9	0.052
$CD4^+FoxP3^-CTLA\text{-}4^+$	2.2 ± 4.2	63.1 ± 95.6	0.004
$CD4^+FoxP3^+CTLA\text{-}4^+$	0.3 ± 0.6	6.1 ± 12.2	0.009
$CD4^+FoxP3^+PD\text{-}L1^+$	22.8 ± 32.3	54.3 ± 53.3	0.015
$CD4^+FoxP3^-PD\text{-}L1^+$	71.9 ± 120.3	264.5 ± 257.6	0.001
$CD68^+$	598.7 ± 436.1	1047.4 ± 643.0	0.013
$CD68^+STING^+$	70.0 ± 117.0	137.1 ± 156.3	0.080
$CD68^+CD163^+HLA\text{-}DR^-$	23.5 ± 42.1	27.3 ± 46.2	0.61

$CD68^+HLA\text{-}DR^+CD163^-$	218.3 ± 154.2	389.1 ± 254.6	0.025
$CD68^+CD163^+HLA\text{-}DR^-STING^+$	7.5 ± 24.6	5.2 ± 12.1	0.15
$CD68^+HLA\text{-}DR^+CD163^-STING^+$	13.1 ± 14.2	38.8 ± 47.5	0.055
$CD20^+$	130.9 ± 160.4	280.6 ± 410.5	0.080
$CD66b^+$	392.1 ± 293.3	656.0 ± 540.9	0.12

[a] Two-sided Mann-Whitney *U* test.

[b] Data presented with Mean ± SD.

Supplementary Table 2. The distribution of tumour-infiltrating immune cells density in tumour core across tumour location

	Tumour location		
	EGJ (N = 24)	Non-EGJ (N = 56)	P value
$CD8^+$	399.9 ± 304.1	385.2 ± 363.3	0.37
$CD8^+PD\text{-}1^+LAG\text{-}3^-$	101.9 ± 86.1	93.9 ± 108.4	0.35
$CD8^+PD\text{-}1^+LAG\text{-}3^+$	17.9 ± 28.0	12.7 ± 20.2	0.33
$CD8^+PD\text{-}1^-LAG\text{-}3^+$	10.5 ± 15.9	15.1 ± 27.0	0.75
$CD8^+PD\text{-}1^-LAG\text{-}3^-$	269.6 ± 228.9	263.6 ± 250.6	0.61
$CD8^+PD\text{-}1^+TIM\text{-}3^+$	25.4 ± 45.9	20.5 ± 44.1	0.45
$CD8^+PD\text{-}1^-TIM\text{-}3^+$	34.8 ± 60.9	39.5 ± 71.3	0.96
$CD8^+PD\text{-}1^+LAG\text{-}3^+TIM\text{-}3^-$	9.5 ± 12.9	7.2 ± 10.5	0.21
$CD8^+PD\text{-}1^+LAG\text{-}3^+TIM\text{-}3^+$	8.4 ± 18.5	5.5 ± 10.8	0.62
$CD8^+PD\text{-}1^+LAG\text{-}3^-TIM\text{-}3^+$	17.0 ± 27.9	15.0 ± 36.2	0.55
$CD8^+PD\text{-}1^+LAG\text{-}3^-TIM\text{-}3^-$	84.9 ± 67.5	78.9 ± 89.5	0.27
$CD4^+$	662.9 ± 379.0	640.3 ± 560.3	0.46
$CD4^+FoxP3^-$	537.1 ± 332.7	507.1 ± 450.7	0.46
$CD4^+FoxP3^+$	125.8 ± 67.5	133.2 ± 125.8	0.65
$CD4^+FoxP3^-CTLA\text{-}4^+$	48.5 ± 75.6	56.3 ± 97.1	0.99
$CD4^+FoxP3^+CTLA\text{-}4^+$	3.4 ± 5.2	5.9 ± 13.2	0.33
$CD4^+FoxP3^+PD\text{-}L1^+$	42.5 ± 34.2	52.6 ± 57.8	0.95
$CD4^+FoxP3^-PD\text{-}L1^+$	210.7 ± 177.4	246.2 ± 277.6	0.75
$CD68^+$	980.1 ± 641.1	980.1 ± 638.1	0.89
$CD68^+STING^+$	115.4 ± 116.7	132.0 ± 166.0	0.46
$CD68^+CD163^+HLA\text{-}DR^-$	33.6 ± 66.0	23.8 ± 33.2	0.68

$CD68^{+}HLA\text{-}DR^{+}CD163^{-}$	387.0 ± 278.2	353.4 ± 237.5	0.79
$CD68^{+}CD163^{+}HLA\text{-}DR^{-}STING^{+}$	4.6 ± 11.3	5.9 ± 15.7	0.35
$CD68^{+}HLA\text{-}DR^{+}CD163^{-}STING^{+}$	35.5 ± 38.1	34.7 ± 48.0	0.54
$CD20^{+}$	323.8 ± 509.4	230.0 ± 321.5	0.56
$CD66b^{+}$	564.1 ± 397.4	638.8 ± 564.6	0.93

[a] Two-sided Mann-Whitney *U* test.

[b] Data presented with Mean ± SD.

Supplementary Table 3. The distribution of tumour-infiltrating immune cells density in tumour core across Lauren classification

	Lauren type			
	Intestinal (N = 38)	Diffuse (N = 18)	Mixed (N = 24)	P value
$CD8^{+}$	302.8 ± 218.1	550.3 ± 517.0	406.6 ± 318.2	0.14
$CD8^{+}PD\text{-}1^{+}LAG\text{-}3^{-}$	77.5 ± 71.5	128.1 ± 151.2	102.0 ± 94.8	0.74
$CD8^{+}PD\text{-}1^{+}LAG\text{-}3^{+}$	15.4 ± 23.6	14.0 ± 22.2	12.7 ± 22.8	0.66
$CD8^{+}PD\text{-}1^{-}LAG\text{-}3^{+}$	11.0 ± 14.8	21.9 ± 39.9	11.8 ± 20.4	0.51
$CD8^{+}PD\text{-}1^{-}LAG\text{-}3^{-}$	198.8 ± 156.5	386.3 ± 348.0	280.1 ± 233.7	0.024
$CD8^{+}PD\text{-}1^{+}TIM\text{-}3^{+}$	21.5 ± 37.3	31.6 ± 67.7	15.5 ± 32.0	0.72
$CD8^{+}PD\text{-}1^{-}TIM\text{-}3^{+}$	30.0 ± 35.6	63.0 ± 110.5	32.4 ± 64.6	0.85
$CD8^{+}PD\text{-}1^{+}LAG\text{-}3^{+}TIM\text{-}3^{-}$	7.9 ± 10.2	8.4 ± 12.2	7.4 ± 12.7	0.80
$CD8^{+}PD\text{-}1^{+}LAG\text{-}3^{+}TIM\text{-}3^{+}$	7.5 ± 15.6	5.6 ± 10.7	5.3 ± 12.1	0.98
$CD8^{+}PD\text{-}1^{+}LAG\text{-}3^{-}TIM\text{-}3^{+}$	14.0 ± 22.2	26.0 ± 59.4	10.2 ± 20.2	0.60
$CD8^{+}PD\text{-}1^{+}LAG\text{-}3^{-}TIM\text{-}3^{-}$	63.5 ± 58.3	102.1 ± 115.0	91.8 ± 86.7	0.62
$CD4^{+}$	677.8 ± 581.4	651.5 ± 540.5	595.3 ± 361.4	0.98
$CD4^{+}FoxP3^{-}$	530.1 ± 464.2	527.4 ± 452.6	485.5 ± 312.5	0.99
$CD4^{+}FoxP3^{+}$	147.7 ± 133.4	124.0 ± 105.0	109.8 ± 68.8	0.55
$CD4^{+}FoxP3^{-}CTLA\text{-}4^{+}$	60.6 ± 94.7	49.8 ± 108.9	46.6 ± 70.5	0.80
$CD4^{+}FoxP3^{+}CTLA\text{-}4^{+}$	6.7 ± 15.0	4.5 ± 8.7	3.4 ± 5.3	0.80
$CD4^{+}FoxP3^{+}PD\text{-}L1^{+}$	54.9 ± 56.1	48.4 ± 59.0	42.2 ± 38.6	0.64
$CD4^{+}FoxP3^{-}PD\text{-}L1^{+}$	244.2 ± 265.0	248.4 ± 287.8	212.3 ± 204.1	0.95
$CD68^{+}$	1005.9 ± 632.6	956.7 ± 682.5	956.9 ± 628.2	0.89
$CD68^{+}STING^{+}$	119.4 ± 116.5	135.6 ± 213.7	132.7 ± 154.8	0.82
$CD68^{+}CD163^{+}HLA\text{-}DR^{-}$	18.1 ± 23.0	30.5 ± 38.8	37.5 ± 69.6	0.47

$CD68^+HLA\text{-}DR^+CD163^-$	391.5 ± 270.7	340.5 ± 248.2	336.4 ± 217.3	0.81
$CD68^+CD163^+HLA\text{-}DR^-STING^+$	2.7 ± 4.7	7.3 ± 16.6	8.7 ± 21.2	0.70
$CD68^+HLA\text{-}DR^+CD163^-STING^+$	38.7 ± 45.1	34.1 ± 55.3	29.6 ± 37.3	0.77
$CD20^+$	260.6 ± 420.9	284.8 ± 319.0	234.4 ± 389.6	0.63
$CD66b^+$	587.5 ± 375.2	742.0 ± 799.3	568.0 ± 457.7	0.86

[a] Kruskal-Wallis Test.

[b] Data presented with Mean ± SD.

Supplementary Table 4. The distribution of tumour-infiltrating immune cells density in tumour core across tumour differentiation

	Tumour differentiation			
	Moderate (N = 23)	Moderate-poor (N = 22)	Poor (N = 35)	P value
$CD8^+$	262.6 ± 182.4	377.3 ± 291.5	480.9 ± 427.3	0.069
$CD8^+PD\text{-}1^+LAG\text{-}3^-$	65.5 ± 70.1	92.8 ± 87.4	118.7 ± 122.4	0.19
$CD8^+PD\text{-}1^+LAG\text{-}3^+$	15.3 ± 26.4	10.7 ± 21.9	15.8 ± 21.1	0.70
$CD8^+PD\text{-}1^-LAG\text{-}3^+$	13.4 ± 16.8	10.5 ± 20.6	15.9 ± 30.1	0.46
$CD8^+PD\text{-}1^-LAG\text{-}3^-$	168.4 ± 126.0	263.3 ± 196.6	330.5 ± 303.6	0.023
$CD8^+PD\text{-}1^+TIM\text{-}3^+$	22.7 ± 44.8	20.7 ± 36.1	22.2 ± 49.7	0.89
$CD8^+PD\text{-}1^-TIM\text{-}3^+$	29.6 ± 34.7	37.3 ± 68.6	44.2 ± 83.4	0.93
$CD8^+PD\text{-}1^+LAG\text{-}3^+TIM\text{-}3^-$	6.9 ± 9.7	5.6 ± 10.7	10.0 ± 12.5	0.61
$CD8^+PD\text{-}1^+LAG\text{-}3^+TIM\text{-}3^+$	8.5 ± 18.2	5.1 ± 12.0	5.9 ± 10.7	0.87
$CD8^+PD\text{-}1^+LAG\text{-}3^-TIM\text{-}3^+$	14.2 ± 26.9	15.7 ± 25.6	16.4 ± 42.2	0.72
$CD8^+PD\text{-}1^+LAG\text{-}3^-TIM\text{-}3^-$	51.2 ± 46.4	77.1 ± 65.7	102.3 ± 104.5	0.18
$CD4^+$	540.2 ± 418.5	720.9 ± 492.1	671.0 ± 574.8	0.42
$CD4^+FoxP3^-$	419.7 ± 335.3	567.7 ± 374.8	547.1 ± 485.2	0.38
$CD4^+FoxP3^+$	120.5 ± 88.6	153.2 ± 144.4	123.9 ± 101.6	0.64
$CD4^+FoxP3^-CTLA\text{-}4^+$	44.1 ± 52.4	62.8 ± 95.7	54.9 ± 107.7	0.83
$CD4^+FoxP3^+CTLA\text{-}4^+$	4.5 ± 5.5	8.2 ± 19.4	3.8 ± 6.6	0.90
$CD4^+FoxP3^+PD\text{-}L1^+$	42.5 ± 40.3	52.2 ± 43.4	52.6 ± 63.1	0.69
$CD4^+FoxP3^-PD\text{-}L1^+$	182.2 ± 157.4	243.3 ± 205.0	265.8 ± 318.8	0.54
$CD68^+$	793.3 ± 544.4	1072.9 ± 614.7	1044.5 ± 691.4	0.25
$CD68^+STING^+$	106.9 ± 120.7	138.0 ± 143.3	133.3 ± 177.3	0.60
$CD68^+CD163^+HLA\text{-}DR^-$	17.4 ± 23.4	36.9 ± 71.3	26.5 ± 34.0	0.79

$CD68^+HLA\text{-}DR^+CD163^-$	329.7 ± 267.3	378.8 ± 232.1	376.1 ± 252.2	0.51
$CD68^+CD163^+HLA\text{-}DR^-STING^+$	2.2 ± 3.5	8.6 ± 21.2	5.8 ± 13.6	0.39
$CD68^+HLA\text{-}DR^+CD163^-STING^+$	35.8 ± 52.6	24.9 ± 19.5	40.6 ± 50.9	0.60
$CD20^+$	165.8 ± 153.0	264.2 ± 526.0	315.1 ± 389.8	0.43
$CD66b^+$	523.9 ± 309.1	637.1 ± 456.1	664.3 ± 653.1	0.84

[a] Kruskal-Wallis Test.

[b] Data presented with Mean ± SD.

Supplementary Table 5. The distribution of tumour-infiltrating immune cells density in tumour core across HER2 expression

	HER2 expression		
	Negative (N = 58)	Positive (N = 22)	P value
$CD8^{+}$	402.6 ± 373.9	355.5 ± 256.8	0.97
$CD8^{+}PD\text{-}1^{+}LAG\text{-}3^{-}$	101.4 ± 110.2	82.7 ± 75.5	0.82
$CD8^{+}PD\text{-}1^{+}LAG\text{-}3^{+}$	13.5 ± 22.4	16.3 ± 24.0	0.41
$CD8^{+}PD\text{-}1^{-}LAG\text{-}3^{+}$	13.5 ± 26.3	14.1 ± 18.2	0.33
$CD8^{+}PD\text{-}1^{-}LAG\text{-}3^{-}$	274.2 ± 262.1	242.4 ± 186.2	0.86
$CD8^{+}PD\text{-}1^{+}TIM\text{-}3^{+}$	22.4 ± 48.7	20.8 ± 31.1	0.44
$CD8^{+}PD\text{-}1^{-}TIM\text{-}3^{+}$	36.1 ± 73.7	43.4 ± 50.7	0.28
$CD8^{+}PD\text{-}1^{+}LAG\text{-}3^{+}TIM\text{-}3^{-}$	7.3 ± 11.2	9.4 ± 11.7	0.42
$CD8^{+}PD\text{-}1^{+}LAG\text{-}3^{+}TIM\text{-}3^{+}$	6.2 ± 13.6	6.9 ± 13.3	0.82
$CD8^{+}PD\text{-}1^{+}LAG\text{-}3^{-}TIM\text{-}3^{+}$	16.2 ± 38.1	13.8 ± 18.6	0.35
$CD8^{+}PD\text{-}1^{+}LAG\text{-}3^{-}TIM\text{-}3^{-}$	85.2 ± 88.8	68.8 ± 66.4	0.81
$CD4^{+}$	609.2 ± 449.8	746.9 ± 645.2	0.45
$CD4^{+}FoxP3^{-}$	486.7 ± 363.7	593.6 ± 535.0	0.54
$CD4^{+}FoxP3^{+}$	122.5 ± 108.1	153.3 ± 118.6	0.20
$CD4^{+}FoxP3^{-}CTLA\text{-}4^{+}$	49.9 ± 88.4	64.6 ± 98.2	0.75
$CD4^{+}FoxP3^{+}CTLA\text{-}4^{+}$	5.3 ± 13.0	4.8 ± 5.9	0.92
$CD4^{+}FoxP3^{+}PD\text{-}L1^{+}$	46.8 ± 45.6	57.0 ± 66.3	0.70
$CD4^{+}FoxP3^{-}PD\text{-}L1^{+}$	216.7 ± 217.2	285.5 ± 325.0	0.53
$CD68^{+}$	923.4 ± 591.5	1129.7 ± 731.2	0.27
$CD68^{+}STING^{+}$	132.5 ± 162.8	112.6 ± 122.8	0.75
$CD68^{+}CD163^{+}HLA\text{-}DR^{-}$	29.7 ± 50.6	18.9 ± 26.4	0.48

CD68$^+$HLA-DR$^+$CD163$^-$	341.3 ± 219.4	422.0 ± 312.8	0.52
CD68$^+$CD163$^+$HLA-DR$^-$STING$^+$	7.0 ± 16.7	1.7 ± 2.8	0.075
CD68$^+$HLA-DR$^+$CD163$^-$STING$^+$	32.6 ± 41.8	41.2 ± 53.1	0.91
CD20$^+$	234.9 ± 319.4	319.5 ± 528.9	0.66
CD66b$^+$	574.4 ± 511.7	727.3 ± 532.6	0.13

[a] Two-sided Mann-Whitney *U* test.

[b] Data presented with Mean ± SD.

Supplementary Table 6. The distribution of tumour-infiltrating immune cells density in tumour core across MMR status

	MMR status		
	pMMR (N = 69)	dMMR (N = 11)	P value
$CD8^{+}$	413.7 ± 362.0	238.7 ± 137.3	0.082
$CD8^{+}PD\text{-}1^{+}LAG\text{-}3^{-}$	100.8 ± 102.5	67.7 ± 95.8	0.13
$CD8^{+}PD\text{-}1^{+}LAG\text{-}3^{+}$	15.5 ± 23.7	6.3 ± 13.3	0.056
$CD8^{+}PD\text{-}1^{-}LAG\text{-}3^{+}$	15.1 ± 25.7	4.6 ± 5.8	0.12
$CD8^{+}PD\text{-}1^{-}LAG\text{-}3^{-}$	282.2 ± 256.6	160.2 ± 62.9	0.13
$CD8^{+}PD\text{-}1^{+}TIM\text{-}3^{+}$	24.7 ± 47.1	5.0 ± 11.5	0.031
$CD8^{+}PD\text{-}1^{-}TIM\text{-}3^{+}$	43.6 ± 71.7	3.8 ± 6.9	0.015
$CD8^{+}PD\text{-}1^{+}LAG\text{-}3^{+}TIM\text{-}3^{-}$	8.4 ± 11.2	4.3 ± 11.6	0.066
$CD8^{+}PD\text{-}1^{+}LAG\text{-}3^{+}TIM\text{-}3^{+}$	7.1 ± 14.3	2.0 ± 5.1	0.069
$CD8^{+}PD\text{-}1^{+}LAG\text{-}3^{-}TIM\text{-}3^{+}$	17.6 ± 35.9	3.0 ± 6.5	0.031
$CD8^{+}PD\text{-}1^{+}LAG\text{-}3^{-}TIM\text{-}3^{-}$	83.3 ± 81.4	64.6 ± 95.9	0.20
$CD4^{+}$	695.7 ± 527.6	342.3 ± 216.0	0.022
$CD4^{+}FoxP3^{-}$	558.7 ± 429.1	249.0 ± 173.9	0.012
$CD4^{+}FoxP3^{+}$	137.0 ± 117.1	93.3 ± 51.5	0.27
$CD4^{+}FoxP3^{-}CTLA\text{-}4^{+}$	62.1 ± 95.3	2.7 ± 4.3	0.076
$CD4^{+}FoxP3^{+}CTLA\text{-}4^{+}$	6.0 ± 12.2	0.3 ± 0.7	0.080
$CD4^{+}FoxP3^{+}PD\text{-}L1^{+}$	51.8 ± 54.3	35.8 ± 31.0	0.57
$CD4^{+}FoxP3^{-}PD\text{-}L1^{+}$	252.0 ± 262.1	132.6 ± 132.4	0.20
$CD68^{+}$	1044.9 ± 657.5	573.8 ± 185.9	0.022
$CD68^{+}STING^{+}$	141.7 ± 158.7	35.2 ± 37.8	0.014
$CD68^{+}CD163^{+}HLA\text{-}DR^{-}$	28.8 ± 48.3	13.5 ± 12.0	0.82

$CD68^{+}HLA\text{-}DR^{+}CD163^{-}$	378.7 ± 261.0	268.4 ± 123.7	0.38
$CD68^{+}CD163^{+}HLA\text{-}DR^{-}STING^{+}$	6.3 ± 15.4	0.8 ± 0.8	0.78
$CD68^{+}HLA\text{-}DR^{+}CD163^{-}STING^{+}$	38.3 ± 47.4	14.0 ± 13.7	0.11
$CD20^{+}$	291.4 ± 406.6	49.5 ± 26.5	0.00021
$CD66b^{+}$	663.2 ± 536.1	323.1 ± 248.4	0.032

[a] Two-sided Mann-Whitney *U* test.

[b] Data presented with Mean ± SD.

Supplementary Table 7. The distribution of tumour-infiltrating immune cells density in tumour core across EBV status

	EBV status		
	Negative ($N = 70$)	Positive (N = 10)	P value
$CD8^+$	342.7 ± 282.5	718.2 ± 542.6	0.011
$CD8^+PD\text{-}1^+LAG\text{-}3^-$	92.7 ± 101.1	121.1 ± 107.7	0.15
$CD8^+PD\text{-}1^+LAG\text{-}3^+$	14.1 ± 23.1	15.4 ± 21.6	0.83
$CD8^+PD\text{-}1^-LAG\text{-}3^+$	10.7 ± 15.5	34.4 ± 52.5	0.23
$CD8^+PD\text{-}1^-LAG\text{-}3^-$	225.1 ± 179.0	547.3 ± 413.6	0.002
$CD8^+PD\text{-}1^+TIM\text{-}3^+$	22.0 ± 46.1	21.6 ± 32.0	0.53
$CD8^+PD\text{-}1^-TIM\text{-}3^+$	34.6 ± 61.1	62.8 ± 105.7	0.49
$CD8^+PD\text{-}1^+LAG\text{-}3^+TIM\text{-}3^-$	7.7 ± 11.2	9.0 ± 12.1	0.98
$CD8^+PD\text{-}1^+LAG\text{-}3^+TIM\text{-}3^+$	6.4 ± 13.8	6.4 ± 11.6	0.87
$CD8^+PD\text{-}1^+LAG\text{-}3^-TIM\text{-}3^+$	15.6 ± 35.4	15.3 ± 20.6	0.39
$CD8^+PD\text{-}1^+LAG\text{-}3^-TIM\text{-}3^-$	77.1 ± 79.1	105.9 ± 109.2	0.22
$CD4^+$	602.7 ± 477.8	957.9 ± 643.3	0.059
$CD4^+FoxP3^-$	483.5 ± 401.2	744.5 ± 475.4	0.081
$CD4^+FoxP3^+$	119.2 ± 89.1	213.3 ± 197.7	0.057
$CD4^+FoxP3^-CTLA\text{-}4^+$	51.9 ± 86.8	68.2 ± 119.7	0.83
$CD4^+FoxP3^+CTLA\text{-}4^+$	4.2 ± 6.3	12.3 ± 27.9	0.88
$CD4^+FoxP3^+PD\text{-}L1^+$	47.2 ± 51.1	66.3 ± 56.4	0.23
$CD4^+FoxP3^-PD\text{-}L1^+$	228.7 ± 256.7	283.5 ± 213.6	0.26
$CD68^+$	951.9 ± 631.0	1177.4 ± 660.3	0.26
$CD68^+STING^+$	115.8 ± 146.7	205.9 ± 175.9	0.061
$CD68^+CD163^+HLA\text{-}DR^-$	24.8 ± 44.9	40.4 ± 48.7	0.19

$CD68^{+}HLA\text{-}DR^{+}CD163^{-}$	367.6 ± 254.6	335.1 ± 216.4	0.88
$CD68^{+}CD163^{+}HLA\text{-}DR^{-}STING^{+}$	4.0 ± 10.6	16.4 ± 28.5	0.23
$CD68^{+}HLA\text{-}DR^{+}CD163^{-}STING^{+}$	34.1 ± 46.7	41.0 ± 32.5	0.083
$CD20^{+}$	254.8 ± 407.6	281.6 ± 192.8	0.13
$CD66b^{+}$	576.8 ± 446.0	893.9 ± 859.7	0.53

[a] Two-sided Mann-Whitney *U* test.

[b] Data presented with Mean ± SD.

Supplementary Table 8. The distribution of tumour-infiltrating immune cells density in tumour core across different CPS score

	CPS				
	<1 (N = 17)	≥1 to 5 (N = 17)	≥5 to 10 (N = 10)	≥10 (N = 36)	P value
$CD8^+$	189.4 ± 169.8	267.7 ± 106.5	366.3 ± 247.4	548.3 ± 425.9	0.000092
$CD8^+PD\text{-}1^+LAG\text{-}3^-$	34.4 ± 41.7	62.5 ± 65.2	106.0 ± 92.5	138.7 ± 118.8	0.000033
$CD8^+PD\text{-}1^+LAG\text{-}3^+$	3.6 ± 5.6	12.6 ± 25.8	16.1 ± 13.7	19.6 ± 26.8	0.008
$CD8^+PD\text{-}1^-LAG\text{-}3^+$	9.1 ± 13.3	5.4 ± 8.5	11.8 ± 11.1	20.3 ± 33.0	0.12
$CD8^+PD\text{-}1^-LAG\text{-}3^-$	142.3 ± 136.3	187.1 ± 72.0	232.4 ± 211.6	369.7 ± 298.1	0.00061
$CD8^+PD\text{-}1^+TIM\text{-}3^+$	5.1 ± 11.3	18.2 ± 44.4	20.6 ± 18.8	32.1 ± 56.1	0.002
$CD8^+PD\text{-}1^-TIM\text{-}3^+$	12.3 ± 22.5	15.1 ± 22.4	28.3 ± 26.2	63.9 ± 92.2	0.008
$CD8^+PD\text{-}1^+LAG\text{-}3^+TIM\text{-}3^-$	1.9 ± 3.3	5.3 ± 9.3	9.1 ± 7.7	11.5 ± 13.9	0.009
$CD8^+PD\text{-}1^+LAG\text{-}3^+TIM\text{-}3^+$	1.6 ± 4.2	7.3 ± 18.5	7.0 ± 8.2	8.0 ± 14.6	0.029
$CD8^+PD\text{-}1^+LAG\text{-}3^-TIM\text{-}3^+$	3.5 ± 7.6	10.9 ± 26.0	13.6 ± 11.4	24.0 ± 45.3	0.0012
$CD8^+PD\text{-}1^+LAG\text{-}3^-TIM\text{-}3^-$	30.9 ± 39.7	51.6 ± 41.5	92.4 ± 95.4	114.7 ± 94.4	0.000059
$CD4^+$	227.9 ± 230.2	479.4 ± 272.4	773.6 ± 607.4	889.1 ± 520.7	0.000002
$CD4^+FoxP3^-$	171.3 ± 184.6	383.2 ± 233.5	594.3 ± 411.9	720.0 ± 444.3	0.000001
$CD4^+FoxP3^+$	56.6 ± 61.5	96.2 ± 66.8	179.2 ± 200.5	169.1 ± 91.5	0.000053
$CD4^+FoxP3^-CTLA\text{-}4^+$	3.6 ± 9.7	28.2 ± 52.7	84.2 ± 108.7	81.6 ± 107.9	0.00015
$CD4^+FoxP3^+CTLA\text{-}4^+$	0.4 ± 0.9	1.9 ± 3.6	14.3 ± 27.2	6.5 ± 7.7	0.00035
$CD4^+FoxP3^+PD\text{-}L1^+$	15.2 ± 23.6	27.4 ± 28.7	55.1 ± 38.1	74.8 ± 59.9	0.000011
$CD4^+FoxP3^-PD\text{-}L1^+$	52.8 ± 80.9	135.9 ± 153.8	265.9 ± 155.5	360.6 ± 291.5	5.23E-7
$CD68^+$	776.2 ± 707.7	667.7 ± 416.3	1098.6 ± 478.5	1191.0 ± 652.3	0.006
$CD68^+STING^+$	48.4 ± 70.3	64.1 ± 75.5	133.9 ± 121.0	192.0 ± 185.7	0.001
$CD68^+CD163^+HLA\text{-}DR^-$	11.8 ± 12.2	11.1 ± 11.8	24.8 ± 22.9	41.7 ± 62.6	0.15

$CD68^{+}HLA\text{-}DR^{+}CD163^{-}$	267.7 ± 178.6	316.4 ± 263.5	370.5 ± 245.9	429.1 ± 261.1	0.13
$CD68^{+}CD163^{+}HLA\text{-}DR^{-}STING^{+}$	0.7 ± 0.9	1.5 ± 3.3	4.6 ± 7.2	10.0 ± 20.3	0.021
$CD68^{+}HLA\text{-}DR^{+}CD163^{-}STING^{+}$	14.2 ± 17.3	22.7 ± 27.9	39.8 ± 37.4	49.2 ± 56.8	0.014
$CD20^{+}$	118.9 ± 150.1	147.0 ± 179.2	264.2 ± 212.9	374.8 ± 520.7	0.005
$CD66b^{+}$	447.6 ± 339.8	406.3 ± 333.9	652.0 ± 602.8	785.5 ± 587.5	0.031

[a] Kruskal-Wallis Test.

[b] Data presented with Mean ± SD.

Supplementary Table 9. The association of immune cells density in tumour core of patients and gastric cancer mortality

	No. of cases	Overall mortality		
		No. of events	Univariable HR (95% CI)	Multivariable HR[a] (95% CI)
$CD8^{+}PD\text{-}1^{+}LAG\text{-}3^{+}TIM\text{-}3^{+}$				
Low	53	38	1 (reference)	1 (reference)
High	27	21	2.05 (1.18, 3.55)	1.98 (1.12, 3.50)
$CD4^{+}FoxP3^{+}CTLA\text{-}4^{+}$				
Low	53	38	1 (reference)	1 (reference)
High	27	21	1.80 (1.04, 3.09)	1.77 (0.98, 3.21)
$CD68^{+}STING^{+}$				
Low	53	38	1 (reference)	1 (reference)
High	27	21	1.73 (1.00, 2.99)	1.83 (1.01, 3.33)

[a] The multivariable Cox regression model initially included stage(I-II vs. III-IV), tumour location (GEJ vs. non-GEJ), tumour differentiation (moderate vs. moderate-poor vs. poor), and ECOG (0 vs. 1). A backward elimination with a threshold of $P = 0.05$ was used to select variables in the final models.

[b] The category of immune cells: High (density ≥ 2/3 of the patients in the study cohort), Low (density < 2/3 of the patients in the study cohort).

Abbreviations: CI, confidence interval; HR, hazard ratio.

Supplementary Table 10. The effective score of tumour-infiltrating immune cells in tumour core across different stages

	Stage		
	I-II (N = 12)	III-IV (N = 68)	P value
$CD8^+$	3.0 ± 2.5	2.3 ± 2.0	0.55
$CD8^+PD\text{-}1^+LAG\text{-}3^-$	3.4 ± 2.6	2.4 ± 2.5	0.12
$CD8^+PD\text{-}1^+LAG\text{-}3^+$	4.2 ± 3.8	3.3 ± 3.6	0.39
$CD8^+PD\text{-}1^-LAG\text{-}3^+$	3.7 ± 3.8	3.1 ± 3.2	0.93
$CD8^+PD\text{-}1^-LAG\text{-}3^-$	2.7 ± 2.5	2.3 ± 2.1	0.76
$CD8^+PD\text{-}1^+TIM\text{-}3^+$	3.4 ± 3.2	2.7 ± 3.2	0.45
$CD8^+PD\text{-}1^-TIM\text{-}3^+$	2.7 ± 2.8	3.1 ± 3.8	0.76
$CD8^+PD\text{-}1^+LAG\text{-}3^+TIM\text{-}3^-$	4.1 ± 4.4	3.2 ± 3.6	0.67
$CD8^+PD\text{-}1^+LAG\text{-}3^+TIM\text{-}3^+$	3.1 ± 3.4	2.3 ± 2.7	0.53
$CD8^+PD\text{-}1^+LAG\text{-}3^-TIM\text{-}3^+$	3.1 ± 3.4	2.5 ± 3.2	0.67
$CD8^+PD\text{-}1^+LAG\text{-}3^-TIM\text{-}3^-$	3.4 ± 2.6	2.4 ± 2.5	0.096
$CD4^+$	2.9 ± 1.8	1.8 ± 1.6	0.010
$CD4^+FoxP3^-$	3.1 ± 2.0	1.9 ± 1.7	0.011
$CD4^+FoxP3^+$	2.8 ± 1.5	2.4 ± 1.9	0.34
$CD4^+FoxP3^-CTLA\text{-}4^+$	2.0 ± 2.3	2.6 ± 2.7	0.27
$CD4^+FoxP3^+CTLA\text{-}4^+$	1.4 ± 1.7	2.0 ± 2.7	0.50
$CD4^+FoxP3^+PD\text{-}L1^+$	2.2 ± 2.4	2.6 ± 2.4	0.35
$CD4^+FoxP3^-PD\text{-}L1^+$	2.4 ± 2.3	2.3 ± 1.9	0.86
$CD68^+$	1.8 ± 1.6	1.5 ± 1.7	0.37
$CD68^+STING^+$	2.3 ± 1.9	2.2 ± 2.0	0.74
$CD68^+CD163^+HLA\text{-}DR^-$	2.8 ± 2.3	2.6 ± 2.2	0.78

$CD68^{+}HLA\text{-}DR^{+}CD163^{-}$	3.2 ± 1.9	2.5 ± 2.1	0.14
$CD68^{+}CD163^{+}HLA\text{-}DR^{-}STING^{+}$	1.1 ± 2.5	2.0 ± 2.5	0.051
$CD68^{+}HLA\text{-}DR^{+}CD163^{-}STING^{+}$	2.4 ± 1.8	2.6 ± 2.5	0.69
$CD20^{+}$	2.1 ± 0.8	1.9 ± 1.4	0.20
$CD66b^{+}$	3.8 ± 2.7	2.8 ± 1.9	0.20

[a] Two-sided Mann-Whitney *U* test.

[b] Data presented with Mean ± SD.

Supplementary Table 11. The effective score of tumour-infiltrating immune cells in tumour core across tumour location

	Tumour location		
	EGJ (N = 24)	Non-EGJ (N = 56)	P value
$CD8^+$	2.1 ± 1.5	2.5 ± 2.3	0.41
$CD8^+PD\text{-}1^+LAG\text{-}3^-$	2.2 ± 1.8	2.7 ± 2.8	0.36
$CD8^+PD\text{-}1^+LAG\text{-}3^+$	2.4 ± 2.2	3.9 ± 4.0	0.13
$CD8^+PD\text{-}1^-LAG\text{-}3^+$	3.2 ± 2.4	3.2 ± 3.6	0.59
$CD8^+PD\text{-}1^-LAG\text{-}3^-$	2.1 ± 1.6	2.5 ± 2.4	0.57
$CD8^+PD\text{-}1^+TIM\text{-}3^+$	2.2 ± 2.1	3.0 ± 3.5	0.34
$CD8^+PD\text{-}1^-TIM\text{-}3^+$	2.4 ± 2.3	3.3 ± 4.1	0.48
$CD8^+PD\text{-}1^+LAG\text{-}3^+TIM\text{-}3^-$	2.3 ± 2.2	3.8 ± 4.2	0.15
$CD8^+PD\text{-}1^+LAG\text{-}3^+TIM\text{-}3^+$	2.1 ± 2.4	2.6 ± 3.0	0.59
$CD8^+PD\text{-}1^+LAG\text{-}3^-TIM\text{-}3^+$	2.0 ± 2.3	2.8 ± 3.5	0.28
$CD8^+PD\text{-}1^+LAG\text{-}3^-TIM\text{-}3^-$	2.2 ± 1.9	2.7 ± 2.8	0.42
$CD4^+$	1.5 ± 1.0	2.1 ± 1.9	0.23
$CD4^+FoxP3^-$	1.6 ± 1.1	2.3 ± 2.0	0.22
$CD4^+FoxP3^+$	1.9 ± 0.9	2.7 ± 2.0	0.13
$CD4^+FoxP3^-CTLA\text{-}4^+$	1.8 ± 1.7	2.8 ± 2.9	0.18
$CD4^+FoxP3^+CTLA\text{-}4^+$	1.3 ± 1.2	2.1 ± 2.9	0.47
$CD4^+FoxP3^+PD\text{-}L1^+$	2.2 ± 1.6	2.7 ± 2.7	0.67
$CD4^+FoxP3^-PD\text{-}L1^+$	2.0 ± 1.4	2.4 ± 2.1	0.40
$CD68^+$	1.4 ± 1.5	1.6 ± 1.7	0.87
$CD68^+STING^+$	2.0 ± 1.7	2.4 ± 2.1	0.68
$CD68^+CD163^+HLA\text{-}DR^-$	2.4 ± 1.8	2.8 ± 2.4	0.66

$CD68^{+}HLA\text{-}DR^{+}CD163^{-}$	2.3 ± 1.4	2.8 ± 2.3	0.56
$CD68^{+}CD163^{+}HLA\text{-}DR^{-}STING^{+}$	1.8 ± 1.8	1.9 ± 2.7	0.51
$CD68^{+}HLA\text{-}DR^{+}CD163^{-}STING^{+}$	2.2 ± 1.8	2.7 ± 2.6	0.67
$CD20^{+}$	1.7 ± 1.3	2.0 ± 1.3	0.37
$CD66b^{+}$	2.6 ± 2.2	3.1 ± 2.0	0.22

[a] Two-sided Mann-Whitney *U* test.

[b] Data presented with Mean ± SD.

Supplementary Table 12. The effective score of tumour-infiltrating immune cells in tumour core across Lauren classification

	Lauren type			
	Intestinal (N = 38)	Diffuse (N = 18)	Mixed (N = 24)	P value
$CD8^+$	2.4 ± 2.4	2.6 ± 1.3	2.3 ± 2.1	0.37
$CD8^+PD\text{-}1^+LAG\text{-}3^-$	2.4 ± 3.0	2.8 ± 1.7	2.6 ± 2.3	0.30
$CD8^+PD\text{-}1^+LAG\text{-}3^+$	3.0 ± 4.0	4.1 ± 2.6	3.7 ± 3.7	0.12
$CD8^+PD\text{-}1^-LAG\text{-}3^+$	3.2 ± 3.9	3.7 ± 2.3	3.0 ± 2.7	0.33
$CD8^+PD\text{-}1^-LAG\text{-}3^-$	2.3 ± 2.5	2.5 ± 1.3	2.4 ± 2.2	0.37
$CD8^+PD\text{-}1^+TIM\text{-}3^+$	2.5 ± 3.6	3.2 ± 2.2	3.0 ± 3.1	0.25
$CD8^+PD\text{-}1^-TIM\text{-}3^+$	3.1 ± 4.7	2.9 ± 2.0	2.9 ± 2.8	0.59
$CD8^+PD\text{-}1^+LAG\text{-}3^+TIM\text{-}3^-$	2.7 ± 4.0	4.2 ± 2.6	3.6 ± 3.9	0.058
$CD8^+PD\text{-}1^+LAG\text{-}3^+TIM\text{-}3^+$	2.1 ± 2.3	3.1 ± 3.1	2.6 ± 3.2	0.64
$CD8^+PD\text{-}1^+LAG\text{-}3^-TIM\text{-}3^+$	2.0 ± 3.6	3.0 ± 2.2	3.1 ± 3.2	0.058
$CD8^+PD\text{-}1^+LAG\text{-}3^-TIM\text{-}3^-$	2.3 ± 3.0	2.8 ± 1.9	2.6 ± 2.4	0.22
$CD4^+$	1.9 ± 2.1	2.1 ± 1.1	2.1 ± 1.2	0.13
$CD4^+FoxP3^-$	2.0 ± 2.3	2.1 ± 1.1	2.1 ± 1.4	0.16
$CD4^+FoxP3^+$	2.3 ± 2.2	2.9 ± 1.5	2.5 ± 1.3	0.058
$CD4^+FoxP3^-CTLA\text{-}4^+$	2.6 ± 3.1	2.5 ± 1.9	2.4 ± 2.4	0.77
$CD4^+FoxP3^+CTLA\text{-}4^+$	1.7 ± 2.0	1.9 ± 2.7	2.2 ± 3.1	0.58
$CD4^+FoxP3^+PD\text{-}L1^+$	2.1 ± 2.5	3.3 ± 2.2	2.7 ± 2.3	0.029
$CD4^+FoxP3^-PD\text{-}L1^+$	2.0 ± 2.1	2.9 ± 1.5	2.4 ± 2.0	0.041
$CD68^+$	1.3 ± 1.6	1.5 ± 1.4	1.8 ± 1.9	0.42
$CD68^+STING^+$	2.2 ± 2.1	2.0 ± 1.2	2.5 ± 2.1	0.62
$CD68^+CD163^+HLA\text{-}DR^-$	2.2 ± 2.0	2.9 ± 1.6	3.2 ± 2.8	0.050

$CD68^{+}HLA\text{-}DR^{+}CD163^{-}$	2.4 ± 2.1	2.6 ± 1.2	3.1 ± 2.4	0.19
$CD68^{+}CD163^{+}HLA\text{-}DR^{-}STING^{+}$	1.7 ± 2.4	1.9 ± 1.7	2.0 ± 3.0	0.55
$CD68^{+}HLA\text{-}DR^{+}CD163^{-}STING^{+}$	2.5 ± 2.7	2.1 ± 1.4	2.9 ± 2.5	0.36
$CD20^{+}$	1.6 ± 1.0	1.9 ± 1.5	2.3 ± 1.4	0.18
$CD66b^{+}$	2.8 ± 2.2	3.0 ± 2.1	3.2 ± 2.0	0.66

[a] Kruskal-Wallis Test.

[b] Data presented with Mean ± SD.

Supplementary Table 13. The effective score of tumour-infiltrating immune cells in tumour core across tumour differentiation

	Tumour differentiation			
	Moderate (N = 23)	Moderate-poor (N = 22)	Poor (N = 35)	P value
$CD8^+$	2.2 ± 1.2	2.9 ± 3.5	2.3 ± 1.2	0.85
$CD8^+PD\text{-}1^+LAG\text{-}3^-$	2.1 ± 1.4	3.2 ± 4.2	2.4 ± 1.4	0.79
$CD8^+PD\text{-}1^+LAG\text{-}3^+$	2.2 ± 1.9	4.1 ± 5.4	3.9 ± 2.9	0.076
$CD8^+PD\text{-}1^-LAG\text{-}3^+$	2.8 ± 2.6	3.8 ± 5.0	3.1 ± 2.1	0.81
$CD8^+PD\text{-}1^-LAG\text{-}3^-$	2.0 ± 1.2	3.0 ± 3.5	2.2 ± 1.3	0.93
$CD8^+PD\text{-}1^+TIM\text{-}3^+$	2.0 ± 1.9	3.7 ± 4.8	2.7 ± 2.4	0.54
$CD8^+PD\text{-}1^-TIM\text{-}3^+$	2.5 ± 2.6	3.8 ± 6.0	2.8 ± 1.9	0.49
$CD8^+PD\text{-}1^+LAG\text{-}3^+TIM\text{-}3^-$	2.0 ± 1.9	4.0 ± 5.7	3.8 ± 2.9	0.051
$CD8^+PD\text{-}1^+LAG\text{-}3^+TIM\text{-}3^+$	1.9 ± 2.3	2.7 ± 3.3	2.7 ± 2.8	0.67
$CD8^+PD\text{-}1^+LAG\text{-}3^-TIM\text{-}3^+$	1.9 ± 1.9	3.2 ± 4.9	2.6 ± 2.4	0.61
$CD8^+PD\text{-}1^+LAG\text{-}3^-TIM\text{-}3^-$	2.1 ± 1.4	3.2 ± 4.2	2.3 ± 1.6	0.88
$CD4^+$	1.5 ± 1.5	2.4 ± 2.3	2.0 ± 1.3	0.13
$CD4^+FoxP3^-$	1.6 ± 1.6	2.5 ± 2.4	2.1 ± 1.5	0.11
$CD4^+FoxP3^+$	1.7 ± 1.3	2.7 ± 2.5	2.8 ± 1.5	0.012
$CD4^+FoxP3^-CTLA\text{-}4^+$	1.6 ± 1.6	3.2 ± 3.4	2.7 ± 2.6	0.12
$CD4^+FoxP3^+CTLA\text{-}4^+$	1.2 ± 1.2	2.9 ± 3.8	1.7 ± 2.0	0.29
$CD4^+FoxP3^+PD\text{-}L1^+$	1.1 ± 0.9	3.3 ± 3.4	3.0 ± 2.0	0.00028
$CD4^+FoxP3^-PD\text{-}L1^+$	1.2 ± 1.0	2.9 ± 2.9	2.6 ± 1.4	0.001
$CD68^+$	1.2 ± 1.6	1.9 ± 1.8	1.5 ± 1.6	0.23
$CD68^+STING^+$	1.8 ± 2.0	2.5 ± 2.2	2.4 ± 1.8	0.096
$CD68^+CD163^+HLA\text{-}DR^-$	1.8 ± 1.5	3.2 ± 2.7	2.8 ± 2.2	0.028

$CD68^+HLA\text{-}DR^+CD163^-$	2.2 ± 1.6	3.1 ± 2.6	2.6 ± 2.0	0.28
$CD68^+CD163^+HLA\text{-}DR^-STING^+$	1.4 ± 1.7	1.9 ± 3.1	2.1 ± 2.4	0.41
$CD68^+HLA\text{-}DR^+CD163^-STING^+$	2.1 ± 2.0	2.8 ± 2.7	2.8 ± 2.4	0.15
$CD20^+$	1.7 ± 1.2	2.4 ± 1.3	1.7 ± 1.3	0.083
$CD66b^+$	2.8 ± 2.6	3.1 ± 1.9	2.9 ± 1.9	0.40

[a] Kruskal-Wallis Test.

[b] Data presented with Mean ± SD.

Supplementary Table 14. The effective score of tumour-infiltrating immune cells in tumour core across HER2 expression

	HER2 expression		
	Negative (N = 58)	Positive (N = 22)	P value
$CD8^+$	2.6 ± 2.3	2.0 ± 1.2	0.43
$CD8^+PD\text{-}1^+LAG\text{-}3^-$	2.7 ± 2.8	2.0 ± 1.6	0.26
$CD8^+PD\text{-}1^+LAG\text{-}3^+$	3.9 ± 4.0	2.3 ± 2.1	0.094
$CD8^+PD\text{-}1^-LAG\text{-}3^+$	3.4 ± 3.5	2.7 ± 2.4	0.55
$CD8^+PD\text{-}1^-LAG\text{-}3^-$	2.6 ± 2.4	1.8 ± 1.3	0.13
$CD8^+PD\text{-}1^+TIM\text{-}3^+$	3.1 ± 3.5	1.9 ± 1.7	0.29
$CD8^+PD\text{-}1^-TIM\text{-}3^+$	3.5 ± 4.1	1.8 ± 1.7	0.041
$CD8^+PD\text{-}1^+LAG\text{-}3^+TIM\text{-}3^-$	3.9 ± 4.1	1.9 ± 2.0	0.040
$CD8^+PD\text{-}1^+LAG\text{-}3^+TIM\text{-}3^+$	2.5 ± 3.0	2.2 ± 2.3	0.90
$CD8^+PD\text{-}1^+LAG\text{-}3^-TIM\text{-}3^+$	3.0 ± 3.6	1.5 ± 1.6	0.099
$CD8^+PD\text{-}1^+LAG\text{-}3^-TIM\text{-}3^-$	2.7 ± 2.8	1.9 ± 1.7	0.14
$CD4^+$	2.1 ± 1.7	1.7 ± 1.8	0.17
$CD4^+FoxP3^-$	2.1 ± 1.8	1.9 ± 2.0	0.19
$CD4^+FoxP3^+$	2.5 ± 1.9	2.3 ± 1.7	0.55
$CD4^+FoxP3^-CTLA\text{-}4^+$	2.6 ± 2.5	2.4 ± 3.1	0.41
$CD4^+FoxP3^+CTLA\text{-}4^+$	2.1 ± 2.8	1.2 ± 1.4	0.25
$CD4^+FoxP3^+PD\text{-}L1^+$	2.9 ± 2.6	1.7 ± 1.6	0.052
$CD4^+FoxP3^-PD\text{-}L1^+$	2.5 ± 2.1	1.7 ± 1.3	0.13
$CD68^+$	1.7 ± 1.7	1.1 ± 1.4	0.15
$CD68^+STING^+$	2.2 ± 1.9	2.3 ± 2.2	0.53
$CD68^+CD163^+HLA\text{-}DR^-$	3.0 ± 2.4	1.7 ± 1.2	0.004

$CD68^+HLA\text{-}DR^+CD163^-$	2.9 ± 2.2	1.9 ± 1.4	0.010
$CD68^+CD163^+HLA\text{-}DR^-STING^+$	2.0 ± 2.7	1.4 ± 1.7	0.30
$CD68^+HLA\text{-}DR^+CD163^-STING^+$	2.6 ± 2.4	2.5 ± 2.4	0.35
$CD20^+$	2.0 ± 1.3	1.7 ± 1.2	0.41
$CD66b^+$	3.0 ± 2.0	2.7 ± 2.4	0.24

[a] Two-sided Mann-Whitney *U* test.

[b] Data presented with Mean ± SD.

Supplementary Table 15. The effective score of tumour-infiltrating immune cells in tumour core across MMR status

	MMR status		
	pMMR (N = 69)	dMMR (N = 11)	P value
$CD8^+$	2.4 ± 2.1	2.3 ± 2.4	0.42
$CD8^+PD\text{-}1^+LAG\text{-}3^-$	2.5 ± 2.5	2.5 ± 2.6	0.74
$CD8^+PD\text{-}1^+LAG\text{-}3^+$	3.3 ± 3.4	4.5 ± 4.7	0.63
$CD8^+PD\text{-}1^-LAG\text{-}3^+$	3.2 ± 3.3	3.2 ± 3.1	0.98
$CD8^+PD\text{-}1^-LAG\text{-}3^-$	2.4 ± 2.1	2.1 ± 2.4	0.23
$CD8^+PD\text{-}1^+TIM\text{-}3^+$	2.8 ± 3.2	2.4 ± 3.0	0.52
$CD8^+PD\text{-}1^-TIM\text{-}3^+$	3.1 ± 3.7	2.1 ± 3.0	0.11
$CD8^+PD\text{-}1^+LAG\text{-}3^+TIM\text{-}3^-$	3.1 ± 3.5	4.6 ± 5.1	0.63
$CD8^+PD\text{-}1^+LAG\text{-}3^+TIM\text{-}3^+$	2.5 ± 2.7	2.1 ± 3.6	0.31
$CD8^+PD\text{-}1^+LAG\text{-}3^-TIM\text{-}3^+$	2.6 ± 3.3	2.2 ± 2.9	0.62
$CD8^+PD\text{-}1^+LAG\text{-}3^-TIM\text{-}3^-$	2.5 ± 2.6	2.5 ± 2.6	0.96
$CD4^+$	1.9 ± 1.7	2.4 ± 1.3	0.12
$CD4^+FoxP3^-$	2.0 ± 1.9	2.5 ± 1.5	0.15
$CD4^+FoxP3^+$	2.5 ± 1.9	2.5 ± 1.2	0.50
$CD4^+FoxP3^-CTLA\text{-}4^+$	2.4 ± 2.7	3.1 ± 2.3	0.21
$CD4^+FoxP3^+CTLA\text{-}4^+$	1.9 ± 2.6	1.7 ± 2.1	0.54
$CD4^+FoxP3^+PD\text{-}L1^+$	2.5 ± 2.5	2.8 ± 1.1	0.12
$CD4^+FoxP3^-PD\text{-}L1^+$	2.2 ± 2.0	2.7 ± 1.5	0.24
$CD68^+$	1.5 ± 1.7	1.7 ± 1.6	0.62
$CD68^+STING^+$	2.3 ± 2.0	1.9 ± 1.5	0.95
$CD68^+CD163^+HLA\text{-}DR^-$	2.7 ± 2.3	2.7 ± 1.3	0.35

CD68$^+$HLA-DR$^+$CD163$^-$	2.6 ± 2.1	2.7 ± 1.6	0.58
CD68$^+$CD163$^+$HLA-DR$^-$STING$^+$	2.0 ± 2.6	0.6 ± 0.6	0.053
CD68$^+$HLA-DR$^+$CD163$^-$STING$^+$	2.7 ± 2.5	2.0 ± 1.7	0.31
CD20$^+$	1.9 ± 1.3	1.7 ± 1.0	0.73
CD66b$^+$	2.8 ± 2.1	3.7 ± 1.7	0.098

[a] Two-sided Mann-Whitney *U* test.

[b] Data presented with Mean ± SD.

Supplementary Table 16. The effective score of tumour-infiltrating immune cells in tumour core across EBV status

	EBV status		
	Negative (N = 70)	Positive (N = 10)	P value
$CD8^+$	2.1 ± 1.5	4.6 ± 3.9	0.004
$CD8^+PD\text{-}1^+LAG\text{-}3^-$	2.2 ± 1.8	5.0 ± 5.0	0.008
$CD8^+PD\text{-}1^+LAG\text{-}3^+$	3.1 ± 2.9	5.6 ± 6.7	0.26
$CD8^+PD\text{-}1^-LAG\text{-}3^+$	2.9 ± 2.5	5.7 ± 6.2	0.070
$CD8^+PD\text{-}1^-LAG\text{-}3^-$	2.0 ± 1.6	4.7 ± 3.9	0.001
$CD8^+PD\text{-}1^+TIM\text{-}3^+$	2.3 ± 2.3	5.8 ± 5.9	0.021
$CD8^+PD\text{-}1^-TIM\text{-}3^+$	2.4 ± 2.4	6.9 ± 7.2	0.002
$CD8^+PD\text{-}1^+LAG\text{-}3^+TIM\text{-}3^-$	3.1 ± 3.0	5.3 ± 6.9	0.44
$CD8^+PD\text{-}1^+LAG\text{-}3^+TIM\text{-}3^+$	2.3 ± 2.8	3.6 ± 3.0	0.18
$CD8^+PD\text{-}1^+LAG\text{-}3^-TIM\text{-}3^+$	2.2 ± 2.4	5.3 ± 6.2	0.12
$CD8^+PD\text{-}1^+LAG\text{-}3^-TIM\text{-}3^-$	2.2 ± 1.8	5.0 ± 5.0	0.008
$CD4^+$	1.8 ± 1.4	3.4 ± 2.9	0.012
$CD4^+FoxP3^-$	1.8 ± 1.5	3.6 ± 3.0	0.010
$CD4^+FoxP3^+$	2.2 ± 1.4	4.2 ± 3.0	0.007
$CD4^+FoxP3^-CTLA\text{-}4^+$	2.3 ± 2.4	3.7 ± 4.0	0.28
$CD4^+FoxP3^+CTLA\text{-}4^+$	1.7 ± 2.4	3.0 ± 3.2	0.13
$CD4^+FoxP3^+PD\text{-}L1^+$	2.2 ± 1.9	5.0 ± 3.7	0.003
$CD4^+FoxP3^-PD\text{-}L1^+$	2.0 ± 1.5	4.2 ± 3.3	0.008
$CD68^+$	1.5 ± 1.5	2.0 ± 2.3	0.62
$CD68^+STING^+$	2.1 ± 1.8	3.4 ± 2.5	0.032
$CD68^+CD163^+HLA\text{-}DR^-$	2.3 ± 1.9	4.8 ± 3.2	0.004

$CD68^+HLA\text{-}DR^+CD163^-$	2.5 ± 1.8	4.0 ± 3.3	0.089
$CD68^+CD163^+HLA\text{-}DR^-STING^+$	1.5 ± 2.0	4.3 ± 3.7	0.002
$CD68^+HLA\text{-}DR^+CD163^-STING^+$	2.3 ± 2.1	4.2 ± 3.4	0.008
$CD20^+$	1.7 ± 1.3	2.9 ± 1.1	0.005
$CD66b^+$	2.9 ± 2.1	3.1 ± 2.2	0.84

[a] Two-sided Mann-Whitney *U* test.

[b] Data presented with Mean ± SD.

Supplementary Table 17. The effective score of tumour-infiltrating immune cells in tumour core across different CPS score

	CPS				
	<1 (N = 17)	≥1 to 5 (N = 17)	≥5 to 10 (N = 10)	≥10 (N = 36)	P value
$CD8^+$	2.1 ± 1.6	2.4 ± 2.1	3.3 ± 4.3	2.3 ± 1.3	0.93
$CD8^+PD\text{-}1^+LAG\text{-}3^-$	1.7 ± 1.7	2.7 ± 2.3	3.8 ± 5.3	2.5 ± 1.6	0.24
$CD8^+PD\text{-}1^+LAG\text{-}3^+$	3.0 ± 3.5	3.4 ± 3.6	5.1 ± 6.7	3.2 ± 2.4	0.74
$CD8^+PD\text{-}1^-LAG\text{-}3^+$	2.9 ± 3.0	3.7 ± 2.9	4.3 ± 6.6	2.8 ± 2.1	0.75
$CD8^+PD\text{-}1^-LAG\text{-}3^-$	1.9 ± 1.4	2.4 ± 2.1	3.4 ± 4.4	2.2 ± 1.5	0.85
$CD8^+PD\text{-}1^+TIM\text{-}3^+$	2.2 ± 2.7	2.2 ± 2.6	4.3 ± 6.1	2.9 ± 2.3	0.26
$CD8^+PD\text{-}1^-TIM\text{-}3^+$	2.5 ± 3.3	2.9 ± 2.6	5.2 ± 7.7	2.7 ± 2.2	0.65
$CD8^+PD\text{-}1^+LAG\text{-}3^+TIM\text{-}3^-$	2.7 ± 3.5	3.6 ± 4.0	4.8 ± 6.9	3.1 ± 2.3	0.63
$CD8^+PD\text{-}1^+LAG\text{-}3^+TIM\text{-}3^+$	1.7 ± 2.9	2.5 ± 3.3	2.5 ± 2.0	2.8 ± 2.8	0.35
$CD8^+PD\text{-}1^+LAG\text{-}3^-TIM\text{-}3^+$	1.7 ± 2.5	2.0 ± 2.5	4.3 ± 6.2	2.8 ± 2.5	0.10
$CD8^+PD\text{-}1^+LAG\text{-}3^-TIM\text{-}3^-$	1.7 ± 1.7	2.7 ± 2.3	3.8 ± 5.2	2.5 ± 1.8	0.28
$CD4^+$	2.7 ± 1.8	1.8 ± 1.0	2.9 ± 3.1	1.4 ± 1.1	0.017
$CD4^+FoxP3^-$	2.9 ± 2.1	1.8 ± 1.1	3.1 ± 3.3	1.5 ± 1.1	0.031
$CD4^+FoxP3^+$	2.9 ± 1.5	2.1 ± 1.2	3.0 ± 3.3	2.3 ± 1.6	0.50
$CD4^+FoxP3^-CTLA\text{-}4^+$	2.9 ± 3.3	2.2 ± 2.1	3.4 ± 4.0	2.3 ± 2.1	0.86
$CD4^+FoxP3^+CTLA\text{-}4^+$	1.5 ± 1.7	1.2 ± 1.4	2.4 ± 3.2	2.3 ± 3.0	0.49
$CD4^+FoxP3^+PD\text{-}L1^+$	2.2 ± 1.9	2.3 ± 2.2	3.4 ± 4.0	2.5 ± 2.2	0.91
$CD4^+FoxP3^-PD\text{-}L1^+$	2.6 ± 1.9	2.3 ± 1.7	3.3 ± 3.6	1.9 ± 1.3	0.62
$CD68^+$	1.9 ± 1.4	1.5 ± 1.7	1.6 ± 2.1	1.3 ± 1.6	0.39
$CD68^+STING^+$	2.4 ± 1.9	2.2 ± 2.0	2.6 ± 2.3	2.1 ± 1.9	0.66
$CD68^+CD163^+HLA\text{-}DR^-$	2.4 ± 1.8	2.3 ± 1.3	4.0 ± 3.1	2.6 ± 2.4	0.34
$CD68^+HLA\text{-}DR^+CD163^-$	3.0 ± 1.4	2.6 ± 1.7	3.4 ± 3.2	2.3 ± 2.1	0.12

$CD68^{+}CD163^{+}HLA\text{-}DR^{-}STING^{+}$	1.3 ± 1.9	0.8 ± 1.1	3.1 ± 3.5	2.2 ± 2.7	0.030
$CD68^{+}HLA\text{-}DR^{+}CD163^{-}STING^{+}$	2.6 ± 2.2	2.3 ± 2.0	3.1 ± 3.4	2.5 ± 2.4	0.80
$CD20^{+}$	1.9 ± 1.2	1.7 ± 1.1	2.0 ± 1.4	2.0 ± 1.4	0.97
$CD66b^{+}$	3.4 ± 2.4	3.1 ± 2.1	2.7 ± 1.8	2.7 ± 2.0	0.85

[a] Kruskal-Wallis Test.

[b] Data presented with Mean ± SD.

Supplementary Table 18. The association of effective density of immune cells in tumour core of patients and gastric cancer mortality

		Overall mortality		
	No. of cases	No. of events	Univariable HR (95% CI)	Multivariable HR[a] (95% CI)
$CD68^{+}STING^{+}$				
Low	60	41	1 (reference)	1 (reference)
High	20	18	1.90 (1.09, 3.32)	1.81 (1.00, 3.28)
$CD68^{+}HLA\text{-}DR^{+}CD163^{-}STING^{+}$				
Low	60	41	1 (reference)	1 (reference)
High	20	18	1.91 (1.10, 3.34)	1.84 (1.03, 3.30)
$CD66b^{+}$				
Low	60	42	1 (reference)	1 (reference)
High	20	17	1.80 (1.02, 3.18)	3.06 (1.63, 5.76)

[a] The multivariable Cox regression model initially included stage (I-II vs. III-IV), tumour location (GEJ vs. non-GEJ), tumour differentiation (moderate vs. moderate-poor vs. poor), and ECOG (0 vs. 1). A backward elimination with a threshold of $P = 0.05$ was used to select variables in the final models.

[b] The category of immune cells: High (density ≥ 3/4 of the patients in the study cohort), Low (density < 2/3 of the patients in the study cohort).

Abbreviations: CI, confidence interval; HR, hazard ratio.

Supplementary Table 19. The ordinal logistic regression analysis to assess the association of immune cells with objective response rate in training cohort of gastric cancer treated with ICI

	N=44	Objective response	
		Univariable OR (95% CI)	Multivariable OR[a] (95% CI)
CD8+PD-1-LAG-3- (density)			
Low [b]	29	1 (reference)	1 (reference)
High	15	0.22 (0.04-1.15)	0.11 (0.01-0.85)
CD68+STING+ (density)			
Low [c]	31	1 (reference)	1 (reference)
High	13	0.55 (0.12-2.41)	0.07 (0.01-0.89)
CD4+FoxP3-PD-L1+ (density)			
Low [d]	18	1 (reference)	1 (reference)
High	26	6.86 (1.30-36.1)	19.51 (1.15-330.7)
CD8+PD-1+LAG-3- (effective score)			
Low [e]	22	1 (reference)	1 (reference)
High	22	6.33 (1.45-27.7)	14.03 (1.62-121.7)

[a] The multivariable ordinal logistic regression model initially included stage(I vs. II vs. III vs. IV), tumour location (GEJ vs. non-GEJ), tumour differentiation (moderate vs. moderate-poor vs. poor), ECOG (0 vs. 1), line of therapy (First line vs. Second line vs. Third line and beyond) and type of anti-PD-1/PD-L1 therapy (monotherapy vs. combination of chemotherapy vs. combination of VEFG-targeted therapy vs. combination of anti-CTLA-4 therapy vs. HER2-targeted therapy). A backward elimination with a threshold of P = 0.05 was used to select variables in the final models. [b-e] The cutoff value of High vs. Low were as follows: 66.7%, 70%, 40%, 50%.

Abbreviations: CI, confidence interval; OR, odds ratio.

Supplementary Table 20. The summary of machine learning models of TIIC signature in validation cohort

Machine learning model	Adjusted parameter			Default parameter		
		95%CI			95%CI	
	Mean	Lower	Upper	Mean	Lower	Upper
Extra trees classifier	0.8034	0.8025	0.8043	0.8108	0.8105	0.8111
Adaboost classifier	0.8541	0.8539	0.8544	0.8001	0.7999	0.8003
Gradient boosting classifier	0.7675	0.7657	0.7692	0.8168	0.8166	0.8171
Multilayer perceptron classifier	0.7458	0.7415	0.7500	0.8372	0.8363	0.8380

Supplementary Table 21. The summary of machine learning models of combined TIIC signature in validation cohort

	Adjusted parameter			Default parameter		
		95%CI			95%CI	
	Mean	Lower	Upper	Mean	Lower	Upper
Extra trees classifier	0.9305	0.9297	0.9314	0.9610	0.9607	0.9613
Adaboost classifier	0.9400	0.9400	0.9400	0.8752	0.8750	0.8755
Gradient boosting classifier	0.8690	0.8667	0.8712	0.8792	0.8791	0.8793
Multilayer perceptron classifier	0.6749	0.6706	0.6792	0.9260	0.9256	0.9264

Supplementary Table 22. The feature importance of four indicator in machine learning models in validation cohort

	CD8+PD-1-LAG-3- (density)			CD68+STING+ (density)			CD4+FoxP3-PD-L1+ (density)			CD8+PD-1+LAG-3- (effective score)		
		95%CI			95%CI			95%CI			95%CI	
	Mean	Lower	Upper	Mean	Lower	Upper	Mean	Lower	Upper	Mean	Lower	Upper
ETC (Default)	0.2169	0.2169	0.2169	0.1898	0.1897	0.1898	0.2681	0.2681	0.2682	0.3252	0.3251	0.3252
ETC (Adjusted)	0.1426	0.1418	0.1433	0.1543	0.1540	0.1546	0.2760	02755	0.2766	0.4271	0.4263	0.4279
ABC (Default)	0.2007	0.2006	0.2009	0.1474	0.1472	0.1475	0.2454	0.2453	0.2455	0.4065	0.4063	0.4067
ABC (Adjusted)	0.2142	0.2141	0.2143	0.0736	0.0731	0.0740	0.3506	0.3500	0.3512	0.3616	0.3614	0.3618
GBC (Default)	0.1993	0.1989	0.1997	0.0685	0.0681	0.0689	0.1795	0.1792	0.1798	0.5527	0.5527	0.5527
GBC (Adjusted)	0.1992	0.1988	0.1996	0.0688	0.0684	0.0692	0.1793	0.1790	0.1796	0.5527	0.5527	0.5528

Abbreviations: ETC, extra tree classifier; ABC, AdaBoost classifier; GBC, gradient boosting classifier.

Supplementary Table 23. The feature importance of seven indicator in machine learning models in validation cohort

	ETC			ABC			GBC		
		95%CI			95%CI			95%CI	
	Mean	Lower	Upper	Mean	Lower	Upper	Mean	Lower	Upper
CD8+PD-1-LAG-3- (density) (Default)	0.1583	0.1583	0.1584	0.1635	0.1633	0.1637	0.1424	0.1420	0.1428
CD8+PD-1-LAG-3- (density) (Adjusted)	0.0912	0.0908	0.0917	0.1690	0.1690	0.1691	0.1426	0.1422	0.1430
CD68+STING+ (density) (Default)	0.1607	0.1606	0.1607	0.1093	0.1091	0.1095	0.0725	0.0722	0.0729
CD68+STING+ (density) (Adjusted)	0.1136	0.1133	0.1139	0.0840	0.0838	0.0841	0.0725	0.0722	0.0729
CD4+FoxP3-PD-L1+ (density) (Default)	0.1981	0.1981	0.1981	0.1886	0.1885	0.1887	0.1266	0.1264	0.1269
CD4+FoxP3-PD-L1+ (density) (Adjusted)	0.1939	0.1936	0.1942	0.2843	0.2841	0.2844	0.1266	0.1263	0.1269
CD8+PD-1+LAG-3- (effective score) (Default)	0.2305	0.2305	0.2306	0.3755	0.3753	0.3757	0.5376	0.5374	0.5377
CD8+PD-1+LAG-3- (effective score) (Adjusted)	0.2792	0.2784	0.2800	0.3135	0.3134	0.3136	0.5376	0.5374	0.5377
MMR status (Default)	0.0457	0.0457	0.0457	0.0130	0.0130	0.0131	3.0E-05	2.9E-05	3.2E-05
MMR status (Adjusted)	0.0916	0.0911	0.0921	0.0242	0.0242	0.0243	3.2E-05	3.0E-05	3.3E-05
EBV status (Default)	0.0515	0.0515	0.0515	0.0148	0.0148	0.0148	0.0282	0.0280	0.0285
EBV status (Adjusted)	0.1400	0.1393	0.1407	0.0640	0.0639	0.0640	0.0282	0.0279	0.0284
CPS score (Default)	0.1552	0.1552	0.1552	0.1354	0.1352	0.1356	0.0926	0.0922	0.0930
CPS score (Adjusted)	0.0905	0.0902	0.0908	0.0611	0.0610	0.0611	0.0925	0.0921	0.0929

Abbreviations: ETC, extra tree classifier; ABC, AdaBoost classifier; GBC, gradient boosting classifier.

Supplementary Table 24. The ordinal logistic regression analysis to assess the association of molecular characterization with objective response rate in gastric cancer treated with ICI

	N=59	Objective response	
		Univariable OR (95% CI)	Multivariable OR[a] (95% CI)
HER2 expression			
Negative	45	1 (reference)	1 (reference)
Positive	14	1.23 (0.35-4.35)	0.63 (0.09-4.46)
MMR status			
pMMR	51	1 (reference)	1 (reference)
dMMR	8	0.26 (0.03-2.30)	0.48 (0.04-5.83)
EBV status			
Negative	50	1 (reference)	1 (reference)
Positive	9	0.22 (0.03-1.92)	0.29 (0.02-3.38)

[a] The multivariable ordinal logistic regression model initially included stage(I vs. II vs. III vs. IV), tumour location (GEJ vs. non-GEJ), tumour differentiation (moderate vs. moderate-poor vs. poor), ECOG (0 vs. 1), line of therapy (First line vs. Second line vs. Third line and beyond) and type of anti-PD-1/PD-L1 therapy (monotherapy vs. combination of chemotherapy vs. combination of VEFG-targeted therapy vs. combination of anti-CTLA-4 therapy vs. HER2-targeted therapy). A backward elimination with a threshold of $P = 0.05$ was used to select variables in the final models.

Abbreviations: CI, confidence interval; OR, odds ratio.

Supplementary Table 25. TIICs-signature and gastric cancer mortality

	No. of cases	Profession-free survival			No. of cases	Overall survival		
		No. of events	Univariable HR (95% CI)	Multivariable HR (95% CI)		No. of events	Univariable HR (95% CI)	Multivariable HR (95% CI)
Validation cohort [a]								
High	10	9	1 (referent)	1 (reference)	10	7	1 (referent)	1 (reference)
Low	6	2	0.08 (0.01-0.62)	0.00 (0.00-0.61)	6	1	0.09 (0.01-0.84)	0.07 (0.01-0.99)

[a] The multivariable Cox regression model initially included MMR status, EBV status and CPS, due to limited sample size. A backward elimination with a threshold of P = 0.05 was used to select variables in the final models.

Abbreviations: CI, confidence interval; HR, hazard ratio.

Supplementary Table 26. Summary of primary antibodies and secondary antibody used for m-IHC

Reagent	Source	Identifier	Dilution
CD8	Cell Signaling Technology	CST70306	1:200
LAG-3	Cell Signaling Technology	CST15372	1:100
TIM-3	Cell Signaling Technology	CST45208	1:500
PD-1	Cell Signaling Technology	CST43248	1:50
PanCK	Cell Signaling Technology	CST4545	1:200
CTLA-4	Abcam	ab227709	1:100
CD4	Biolynx	BX22300130	1:6000
FoxP3	Biolegend	BLG320202	1:50
PD-L1	Cell Signaling Technology	CST13684	1:100
STING	Cell Signaling Technology	CST13647	1:1000
CD68	ZSGB-BIO	ZM0060	1:1
HLA-DR	Abcam	ab92511	1:1200
CD163	Cell Signaling Technology	CST93498	1:1000
CD66b	Gene Tex	GTX19779	1:300
CD20	Abcam	ab78237	1:3000
Horseradish-peroxidase-conjugated anti-rabbit and anti-mouse antibody	Biolynx	BX10001	1:3

Supplementary Table 27. The parameters adjusted in machine learning models

Classifiers	Parameters	Codes for the range of parameter selection in Python
Extra tree classifier	n_estimators	'n_estimators': range (1000, 5001, 1000)
	criterion	'criterion': ['gini','entropy']
	max_depth	'max_depth': range (1, 10)
Adaboost classifier	n_estimators	'n_estimators': range (1000, 5001, 1000)
	algorithm	'algorithm': ['SAMME', 'SEMMA.R']
Gradient boosting classifier	n_estimators	'n_estimators': range (1000, 5001, 1000)
Multilayer perceptron	hidden_layer_sizes	[(n*20,)* m form in range (2, 6) for n in range(1, 11)]
	alpha	np.logspace (-4, 3, 8, base = 10)
