# Supplementary material for: Predicting response to immunotherapy in gastric cancer via multi-dimensional analyses of the tumour immune microenvironment
Source: Nat Commun. 2022 Aug 18;13:4851. doi: 10.1038/s41467-022-32570-z (PMC9388563; doi:10.1038/s41467-022-32570-z)
Supplement: Supplementary file 3 — Reporting Summary [file 41467_2022_32570_MOESM3_ESM.pdf]

nature portfolio

Corresponding author(s): Lin Shen
Last updated by author(s): 12/7/2022

Reporting Summary

Nature Portfolio wishes to improve the reproducibility of the work that we publish. This form provides structure for consistency and transparency in reporting. For further information on Nature Portfolio policies, see our Editorial Policies and the Editorial Policy Checklist.

Statistics

For all statistical analyses, confirm that the following items are present in the figure legend, table legend, main text, or Methods section.

n/a	Confirmed	
☐	☒	The exact sample size (*n*) for each experimental group/condition, given as a discrete number and unit of measurement
☐	☒	A statement on whether measurements were taken from distinct samples or whether the same sample was measured repeatedly
☐	☒	The statistical test(s) used AND whether they are one- or two-sided<br>*Only common tests should be described solely by name; describe more complex techniques in the Methods section.*
☒	☐	A description of all covariates tested
☐	☒	A description of any assumptions or corrections, such as tests of normality and adjustment for multiple comparisons
☐	☒	A full description of the statistical parameters including central tendency (e.g. means) or other basic estimates (e.g. regression coefficient) AND variation (e.g. standard deviation) or associated estimates of uncertainty (e.g. confidence intervals)
☐	☒	For null hypothesis testing, the test statistic (e.g. *F*, *t*, *r*) with confidence intervals, effect sizes, degrees of freedom and *P* value noted<br>*Give P values as exact values whenever suitable.*
☒	☐	For Bayesian analysis, information on the choice of priors and Markov chain Monte Carlo settings
☒	☐	For hierarchical and complex designs, identification of the appropriate level for tests and full reporting of outcomes
☐	☒	Estimates of effect sizes (e.g. Cohen's *d*, Pearson's *r*), indicating how they were calculated

Our web collection on statistics for biologists contains articles on many of the points above.

Software and code

Policy information about availability of computer code

Data collection	The multispectral images were analyzed using the inForm image analysis software 2.4 (PerkinElmer). The inForm software actively learned the phenotyping algorithm from all spectrally unmixed images. Each DAPI-stained cell was individually identified according to its combination of fluorophore characteristics and cell morphology features associated with a segmented nucleus (DAPI signal). The density of cells in each ROI was calculated via the normalization of the total cell counts by the total area (cell/ mm2).
Data analysis	The relationships between TIICs and clinicopathologic features were evaluated using the Mann–Whitney U test, the Kruskal–Wallis test, or Pearson correlation analyses, as appropriate. For multiple comparisons of immune cell density and tumor location (TC, IM, N), we used Dunn's adjustment. We also used the Kaplan–Meier method to estimate survival functions and the log-rank test to compare survival distributions. We conducted logistic regression analyses to examine the association of TIICs or molecular features of GC with treatment response. The assumption of proportionality of hazards was assessed by a time-varying covariate in the Cox models with a cross-product term of survival time and each TIIC. The proportional hazard assumptions were generally satisfied for survival (P > 0.05). Additionally, to disclose the potential relationship between each TIIC and GC survival, multivariable-adjusted Cox proportional hazards regression analysis was used. All statistical analyses were performed using SPSS 22.0 (IBM, Armonk, NY) and GraphPad Prism 7.0 (GraphPad Software, San Diego, CA). All P-values were two-tailed.<br>The supervised classifiers in this research were constructed using scikit-learn (version 0.23.2), one of the most popular machine learning programs in Python. Four types of ensemble classifiers were built, including the extra tree classifier, gradient boosting classifier, AdaBoost classifier, and multilayer perceptron.

For manuscripts utilizing custom algorithms or software that are central to the research but not yet described in published literature, software must be made available to editors and reviewers. We strongly encourage code deposition in a community repository (e.g. GitHub). See the Nature Portfolio guidelines for submitting code & software for further information.

March 2021

Data

Policy information about availability of data

All manuscripts must include a data availability statement. This statement should provide the following information, where applicable:

- Accession codes, unique identifiers, or web links for publicly available datasets
- A description of any restrictions on data availability
- For clinical datasets or third party data, please ensure that the statement adheres to our policy

All data relevant to the study are included in the article or uploaded as supplementary information/source data file. The original data will be available on reasonable request.

Field-specific reporting

Please select the one below that is the best fit for your research. If you are not sure, read the appropriate sections before making your selection.

☒ Life sciences ☐ Behavioural & social sciences ☐ Ecological, evolutionary & environmental sciences

For a reference copy of the document with all sections, see nature.com/documents/nr-reporting-summary-flat.pdf

Life sciences study design

All studies must disclose on these points even when the disclosure is negative.

Sample size	No sample size calculation was performed. The 80 samples used in this study were selected which were representative of GC cancer subtypes, with detailed clinical information, and with sufficient qualified tissues. Multiplex immunohistochemistry was performed to visualize the expression of CD8, PD-1, TIM-3, LAG-3, CD4, FoxP3, CTLA-4, PD-L1, CD68, CD163, HLA-DR, STING, CD20, and CD66b on 80 gastric cancer patients , including 60 patients receiving immunotherapy which is for now a relatively large population compared with previous studies.
Data exclusions	Multispectral imaging data were subjected to quality control (QC) by a pathologist, with the subsequent exclusion of the inappropriate regions from the analysis as well as the confirmation of outlier results.
Replication	No replication was included on individual patient specimens given limited tissue availability. We split patients into training cohort and validation cohort to verify the predict value of multi-dimensional tumor infiltrating immune cell-signature. We used four types of machine learning models and calculated the area under the curve of the training and validation cohorts, thus excluding reproducibility issues.
Randomization	No randomization of patients was required in the study. All 60 patients who received immunotherapy were assigned to the training (n=44, generated retrospectively from 2016/11/15 to 2019/7/17) and validation (n=16, generated prospectively from 2019/7/29 to 2019/12/19) cohorts.
Blinding	Two specialized pathologists (blinded to the patient's information) evaluated all GC specimens. Sample classification and data analysis, were all via unsupervised or blinded approaches. The investigators were not blinded afterwards to perform analyses of comparing different clinical parameters to determine their biological relevance.

Reporting for specific materials, systems and methods

We require information from authors about some types of materials, experimental systems and methods used in many studies. Here, indicate whether each material, system or method listed is relevant to your study. If you are not sure if a list item applies to your research, read the appropriate section before selecting a response.

Materials & experimental systems

n/a	Involved in the study	
☐	☒	Antibodies
☒	☐	Eukaryotic cell lines
☒	☐	Palaeontology and archaeology
☒	☐	Animals and other organisms
☐	☒	Human research participants
☒	☐	Clinical data
☒	☐	Dual use research of concern

Methods

n/a	Involved in the study	
☒	☐	ChIP-seq
☒	☐	Flow cytometry
☒	☐	MRI-based neuroimaging

Antibodies

Antibodies used	Multiplex IHC: CD8 (Cell Signaling Technology, CST70306), LAG-3 (Cell Signaling Technology, CST15372), TIM-3 (Cell Signaling Technology, CST45208), PD-1 (Cell Signaling Technology, CST43248), CTLA-4 (Abcam, ab227709), CD4 (Biolynx, BX22300130), FoxP3

	(Biolegend, BLG320202), PD-L1 (Cell Signaling Technology, CST13684), STING (Cell Signaling Technology, CST13647), CD68 (ZSGB-BIO, ZM0060), HLA-DR (Abcam, ab92511), CD163 (Cell Signaling Technology, CST93498), CD66b (Gene Tex, GTX19779), CD20 (Abcam, ab78237), and Pan-cytokeratin (Cell Signaling Technology, CST4545). Horseradish-peroxidase-conjugated anti-rabbit and anti-mouse (Biolynx, BX10001).
Validation	Multiplex IHC: All antibodies were for human. All primary antibodies were optimized using the positive control tissues as recommended by the manufacturers.

Human research participants

Policy information about studies involving human research participants

Population characteristics	Eighty patients were included in this study between July 2014 and December 2019. The median age of the patients was 60 years (range, 54-66 yr), and the majority of patients were men (76.3%). Among the 60 patients subjected to immunotherapy. Archived pre-treatment samples were available from all patients. Ten (12.5%) patients were EBV (+) and 11 (13.75%) had confirmed dMMR GC. See Table 1.
Recruitment	Formalin-fixed and paraffin-embedded (FFPE) GC tissues were obtained from the Department of Pathology, Peking University Cancer Hospital. GC tissues included 80 samples with histologically confirmed gastric adenocarcinoma. We excluded patients with concurrent autoimmune diseases, HIV, or syphilis.
Ethics oversight	This study was approved by the Ethics Committee of the Peking University Cancer Hospital.

Note that full information on the approval of the study protocol must also be provided in the manuscript.
